# Impact of WHO AWaRe Antibiotic Handbook training on antibiotics prescribing knowledge among private primary care providers: a vignette-based, prep–post pilot study in Patna, India

**DOI:** 10.1186/s13756-026-01735-6

**Published:** 2026-03-27

**Authors:** Poshan Thapa, Prachi Shukla, Chandrashekhar Joshi, Sena Sayood, Pradeep Kumar Sinha, Diwash Timilsina, Mili Dutta, Madhukar Pai, Samira Abbasgholizadeh Rahimi, Sumanth Gandra

**Affiliations:** 1 School of Population and Global Health, McGill University, Montreal, QC Canada; 2 World Health Partners, New Delhi, Delhi India; 3 Washington University School of Medicine, St. Louis, USA; 4 Swansea University, Swansea, UK; 5 Department of Family Medicine, McGill University, Montreal, QC Canada

**Keywords:** Primary care providers, WHO AWaRe Antibiotic Handbook, Informal healthcare providers, Training, Pre–post study, Pilot study, India

## Abstract

**Introduction:**

Inappropriate antibiotic prescribing is a major concern in low- and middle-income countries (LMICs), particularly at the primary care level. The WHO AWaRe Antibiotic Handbook was introduced to promote rational antibiotic use, yet its real-world feasibility and potential impact remain underexplored. Our study evaluated the impact and usefulness of the WHO AWaRe Handbook training among private primary care providers (PCPs) in Patna, India.

**Methods:**

We conducted a pre–post pilot study among 145 private PCPs (40 formal PCPs (FPs) and 105 informal PCPs (IPs) in Patna, India. Participants received training from an infectious disease physician on the WHO AWaRe Antibiotic Handbook. Antibiotic prescribing knowledge was assessed before and after the intervention using clinical vignettes for four conditions: acute diarrhea, urinary tract infection (UTI), cellulitis, and community-acquired pneumonia (CAP). An endline survey evaluated the perceived usefulness of the intervention. Changes in prescribing knowledge was analyzed using McNemar’s test for paired data.

**Results:**

The intervention significantly reduced overall antibiotic prescribing knowledge for acute diarrhea (*p* = 0.0003) and UTI (*p* = 0.0113), with greater reductions among IPs. No significant changes were observed for cellulitis (*p* = 0.3692) or CAP (*p* = 0.7150). Watch-category antibiotic prescribing significantly decreased for acute diarrhea (*p* < 0.0001), with no significant changes for other conditions. IPs showed greater improvements overall compared to FPs. The majority of PCPs (75%; n = 107) rated the training as moderately or very useful.

**Conclusion:**

Training private PCPs using the WHO AWaRe Handbook improved antibiotic prescribing knowledge for some common conditions, particularly among IPs. Future research should combine training with strategies that address broader contextual barriers, alongside tailored reinforcement interventions, and longer-term follow-up.

**Supplementary Information:**

The online version contains supplementary material available at 10.1186/s13756-026-01735-6.

Strengths and limitations of this study

- This is the first study to evaluate the impact and usefulness of the WHO AWaRe Handbook training on improving antibiotic prescribing knowledge among private primary care providers (PCPs) in India, focusing on both formal PCPs (FP) and informal PCPs (IPs).
- Using a vignette-based, pre–post design allowed for standardized assessment of prescribing knowledge across four common clinical conditions: acute diarrhea, cellulitis, community-acquired pneumonia, and urinary tract infection.
- Stratified analysis by provider type offered important insights into the intervention's differential effects, particularly highlighting knowledge improvements among IPs.
- While the study captures shifts in prescribing knowledge, it does not assess actual prescribing behaviour in clinical practice, which may limit the generalizability of the findings.
- The study evaluated outcomes over a short follow-up period, which restricts understanding of the sustainability of intervention effects over time.

## Introduction

Global antibiotic use increased 16.3% (2016–2023), mainly in low- and middle-income countries (LMICs) (+ 18.6%), while high-income countries saw a 4.9% decline [1]. Overuse and misuse of antibiotics remain a significant challenge, especially at the primary care level, where over 50% of outpatient visits in LMICs result in an antibiotic prescription, often without clinical justification [2]. A secondary analysis using the data collected through the standardized patient method, a well-established approach for assessing real-world provider practice, documented high rates of inappropriate prescribing in primary care settings across three LMICs (India, China and Kenya). Broad-spectrum antibiotics were excessively used in China and India [3]. The COVID-19 pandemic further exacerbated antibiotic overuse globally, against a backdrop of steadily increasing antibiotic consumption over preceding decades, including a 103% rise in India between 2000 and 2015, which made India the largest consumer of antibiotics worldwide [4]. The challenge of antibiotic over-consumption is particularly complex in India due to its fragmented healthcare system, which includes a range of formal and informal healthcare providers, many of whom prescribe antibiotics empirically, often without regulatory oversight [3, 5]. Antibiotic dispensing among informal healthcare providers is driven by a range of interacting intrinsic and extrinsic factors, and evidence from primary studies conducted in India and reviews focused on LMICs consistently identifies limited knowledge and misconceptions about antibiotics and their use as one of the most common drivers of this practice [6–8].

In India, primary health care within the government’s formal system is predominantly provided through primary health centres (PHCs) and their subsidiary sub-centres (SCs) [9]. However, India’s health system also includes a large private sector, which provides approximately 87% of first-contact primary care, particularly in underserved regions. The private health sector comprises a spectrum of providers, from small clinics or practitioner-run setups to multispecialty hospitals. Primary care providers (PCPs) vary widely in training, ranging from formally qualified practitioners to non-qualified informal healthcare providers with varying educational backgrounds [10, 11]. Formal private PCPs (FPs) typically hold Bachelor of Medicine, Bachelor of Surgery (MBBS) degrees, whereas informal PCPs (IPs), commonly called rural medical practitioners or village doctors, operate outside the formal health system and lack accredited medical qualifications. Despite this, they frequently dispense allopathic treatments, including antibiotics and injections, without formal training [11–13]. Additionally, practitioners of Ayurveda, Yoga & Naturopathy, Unani, Siddha, and Homeopathy (AYUSH) are known to prescribe and dispense antibiotics in India [14], despite not being legally authorized to prescribe modern medicine, including antibiotics.

Considering the need for global antibiotic stewardship, WHO introduced the AWaRe (Access, Watch, Reserve) classification system in 2017. This framework emphasizes the preferential use of narrow-spectrum “Access” antibiotics over broad-spectrum “Watch” and last-resort “Reserve” antibiotics, with WHO recommending that at least 60% of all antibiotic use should consist of Access-group antibiotics [15]. In November 2022, WHO expanded this initiative by publishing the AWaRe Antibiotic Handbook, a clinical decision-support tool providing evidence-based guidance on antibiotic necessity, choice, dosage, route, and duration [16].

Although the AWaRe Handbook is a promising tool for antimicrobial stewardship (AMS), challenges related to the uptake and integration of guideline-based clinical decision-support tools have been reported across diverse health system contexts. Studies indicate that such tools are often underutilized unless adapted to local contexts and effectively delivered to PCPs [17, 18]. To date, research evaluating AWaRe-aligned educational interventions remains sparse, with one hospital-based study from Jordan reporting improvements in provider knowledge and shifts toward greater use of Access-group antibiotics following an educational intervention [19]. Related evidence from China suggests that simplified, guideline-based decision-support approaches, such as a traffic-light clinical guideline, reduced antibiotic prescribing from 82 to 40% in the intervention group, compared with a 5-percentage-point reduction in the control group [20]. However, similar evidence is lacking in the context of India, where overprescription of broad-spectrum antibiotics remains prevalent. Furthermore, translation into regional languages and integration into existing clinical workflows may be necessary to enhance uptake among private PCPs, especially IPs, who may have limited access to standardized treatment guidelines.

Therefore, we conducted a pre–post pilot study using the WHO AWaRe Handbook to evaluate the impact of training private PCPs in Patna, India, on antibiotic prescribing knowledge and the usefulness of the intervention.

## Methods

### Design

We conducted a pre–post pilot study in Patna, India, to assess changes in antibiotic prescribing knowledge among private PCPs before and after a training intervention. Similar approaches have been used in studies with comparable objectives [21–23]. The study was conducted in collaboration with World Health Partners (WHP), a local non-governmental organization (NGO) with over a decade of experience working with the private healthcare sector across various states and districts in India, including Patna.

### Study site

Our study was conducted in Patna, the capital and largest city of Bihar, which serves as the district's administrative headquarters. As per the 2011 census, the urban agglomeration had a population of 2.05 million [24]. Patna was selected due to WHP's ongoing work and collaborations, which facilitated field implementation and participant enrollment, making it an operationally feasible location.

### Study population and sampling

The study included private PCPs offering first-contact consultation services through independent clinics in Patna, India. In this study, private PCPs were defined as providers who routinely manage common, uncomplicated health conditions at the community level and serve as the initial point of care for patients in the private sector. Both FPs and IPs were enrolled. AYUSH providers were pragmatically grouped with IPs to align with prescribing regulations in India, as neither group is authorized to prescribe modern medicine, including antibiotics. The study focused on private PCPs because evidence from primary health care settings in India indicates a high prevalence of antibiotic prescribing, with a pooled estimate of 65% across more than 28,000 patient encounters [25]. In addition, national private-sector data from a 12-month medical audit dataset (May 2013 to April 2014) reported approximately 519 million antibiotic prescriptions annually [26], alongside high antibiotic dispensing by IPs [5], supporting the focus on private PCPs in this study. Convenience sampling was used to recruit PCPs across Patna city using WHP's existing network. PCPs' recruitment covered clinics located across 75 wards, the smallest administrative unit in India. WHP field officers, who routinely engage with private PCPs for various project activities, compiled a list of eligible participants. For IPs, snowball sampling was additionally employed, with enrolled IPs referring eligible peers. PCPs were invited through in-person visits or phone calls, and those who provided written informed consent were enrolled. Of the 325 PCPs (130 FPs and 195 IPs) approached, 146 (45%) consented to participate. Of these, 145 PCPs (40 FPs and 105 IPs) attended the training and completed both the baseline and follow-up vignettes. The development of the sampling frame and provider recruitment was conducted between October and November 2023.

### Sample size

As this was a pilot study, the sample size was based on feasibility and available resources. The final sample of 145 PCPs aligns with recommendations suggesting 100–120 participants for pilot studies to estimate key parameters [27]. The use of a pre–post design further reduced the variability. While sufficient for exploratory analysis, the findings should be interpreted as preliminary to guide future larger-scale studies.

### Description of intervention

All participating PCPs received in-person training on the WHO AWaRe Antibiotic Handbook, delivered by a US board-certified infectious disease physician (SG) who was also trained in India and contributed to the development of the Handbook. The AWaRe Antibiotic Handbook was selected because it explicitly integrates antimicrobial stewardship principles into practical clinical guidance, providing condition-specific recommendations on symptom assessment, diagnosis, the need for antibiotics, and, where indicated, appropriate choice, dose, duration, and frequency. Its structured and accessible format makes it relevant to both FPs and IPs, and its status as a WHO-endorsed guideline enhances its wider acceptability and uptake across diverse care settings. The training was conducted in two phases: 53 attendees (14 FPs and 39 IPs) in December 2023 and 93 attendees (26 FPs and 67 IPs) in March 2024 (a total of 146 PCPs attended; one provider did not complete the endline survey and was excluded from the study) at locations selected for accessibility and participation. Training sessions were conducted separately for FPs and IPs, typically lasting 3 to 4 h each, to accommodate differences in language preferences, baseline clinical knowledge, and to minimize power dynamics that could affect engagement during training. The training covered an overview of antibiotics, antimicrobial resistance, rational prescribing, the AWaRe classification, and its application. Case-based discussions were conducted for four common conditions associated with widespread antibiotic use: acute diarrhea, acute pharyngitis, community-acquired pneumonia (CAP), and lower urinary tract infection (UTI).

Participants were encouraged to ask questions, and discussions addressed field-level prescribing challenges and AWaRe guidelines. PCPs were also introduced to the AWaRe Handbook’s Mobile and Web Application, assisted in downloading it, and provided a tutorial on its use. At the end of the training, FPs received a hard copy of the AWaRe Handbook (English), and IPs received Hindi-translated infographics (provided as supplementary file S1) covering eight conditions: acute bronchitis, acute diarrhea, acute otitis media, acute pharyngitis, acute sinusitis, cellulitis, CAP and UTI. The infographics were adapted from the WHO AWaRe Antibiotic Handbook and translated into Hindi in-house by the WHP team. The translated materials were reviewed by an infectious disease physician (SG), a member of the study team who is proficient in Hindi, to ensure accuracy and contextual relevance for IPs who were not comfortable with English-language materials. To ensure consistency in this study’s clinical vignettes, the term “prescribing” was used for both FPs and IPs, though IPs typically “dispense” rather than “prescribe” antibiotics.

### Outcome and measurement

Provider knowledge was assessed using clinical vignettes, a well-established method for evaluating changes in provider knowledge and clinical decision-making in antibiotics research [28–30]. The vignettes were developed through discussions among co-authors, led by infectious disease physicians (SG and SS), and adapted from validated vignettes used in previous studies [31, 32]. The final set of vignettes (Table 1) was pilot tested with 10 PCPs (excluded from the final analysis), and feedback indicated that providers understood the case scenarios well. For each vignette, PCPs reported whether they would prescribe an antibiotic and, if applicable, specified the antibiotic(s); providers could also include additional details such as dose and duration when completing the vignettes. To inform vignette selection, one infectious disease specialist study team member (SG) conducted a field visit to Patna in May 2023 and consulted seven PCPs to identify common conditions for which antibiotics are prescribed in routine practice. Providers most frequently cited respiratory tract infections (RTI), diarrhoea, fever, skin and soft tissue infections, and UTI. Based on these consultations, eight clinical vignettes were initially developed. However, during the final pretesting of the study tools, providers raised concerns regarding questionnaire length and completion time, leading to a reduction to four vignettes: acute diarrhea, cellulitis, mild community-acquired pneumonia (CAP), and UTI. Approximately 5% of providers completed fewer than four vignettes. These conditions were selected to represent respiratory, gastrointestinal, urinary, and skin and soft tissue infections and aligns with both field consultations and existing evidence showing that antibiotics are commonly prescribed in India’s private sector for diarrhoeal illness, RTI, and UTI [5, 25, 33].

**Table 1:** Vignette cases and antibiotic prescription in concordance with the WHO AWaRe Antibiotic Handbook

Cases	Case description	Antibiotic prescription in concordance with WHO AWaRe Handbook
Cellulitis	A 40-year-old man presents to you with pain and redness in his right leg. It started 3 days ago when he noticed pain and redness in his right foot along with fever. The redness and pain extended to the middle of the right leg. He denies nausea or vomiting. He is otherwise healthy. His vital signs are normal except for a temperature of 100.5 F. On physical examination, there is redness extending from the middle of the right leg to the foot along with warmth and tenderness on palpation. He has no drug allergies	*Concordant*: Prescription of Access-group antibiotics only *Not-concordant*: no antibiotics prescribed, or Watch/Reserve antibiotics prescribed (alone or in combination)
Acute Diarrhea	A 5-year-old boy presents with diarrhea that began 2 days ago. According to the boy’s mother, the diarrhea is watery and yellow and does not have mucus or blood in it. The mother reports no fever, abdominal pain, nausea, or vomiting. He is otherwise a healthy child. Her vital signs and physical examination are normal. There are no signs of dehydration. He has no drug allergies	*Concordant*: No antibiotics are required *Not-concordant*: any antibiotic is prescribed (Access, Watch, or Reserve)
UTI (Urinary Tract Infection)	A 8-year-old girl presents with 2 days of pain while urinating. According to the child’s mother, symptoms started 2 days ago with pain on urination, increased frequency, and urgency. The mother reports no fever, nausea, vomiting or vaginal discharge. She is otherwise a healthy child. Her vital signs and physical examination are normal except for tenderness in the suprapubic area on palpation. She has no drug allergies	*Concordant*: Prescription of Access-group antibiotics only *Not-concordant*: no antibiotics prescribed, or Watch/Reserve antibiotics prescribed (alone or in combination)
CAP (Community Acquired Pneumonia)	A 35-year-old woman presents with 4 days of cough with yellow, non-bloody sputum. Her symptoms started 4 days ago with a runny nose and fever. She continues to have a fever with chills every day. She denies any sick contacts. She is otherwise healthy. Her vital signs are normal except for a temperature of 100.8°F. Upon physical examination, she had a few crackles at the left base of her lungs. Chest X-ray is notable for an infiltrate in the left lower lobe. She has no drug allergies	*Concordant*: Prescription of Access-group antibiotics only *Not-concordant*: no antibiotics prescribed, or Watch/Reserve antibiotics prescribed (alone or in combination)

Since the primary aim of this study was not to assess case management accuracy (such as history-taking, laboratory workup and final diagnosis), results are presented based on changes and shifts in antibiotic prescribing patterns for the four vignette conditions before and after the intervention. The primary outcome was the change in overall antibiotic prescriptions and Watch-category antibiotic use for each vignette condition. The secondary outcome measures were changes in the proportion of antibiotic prescriptions and the concordance of antibiotic prescriptions per the AWaRe Handbook for each clinical condition, as described in Table 1.

During data collection, interviewers first recorded the participants' socio-demographic information. PCPs then completed the vignettes in printed paper format, which were self-administered unless assistance was requested from field officers, which occurred in a few cases. The vignettes were primarily administered at provider clinics by WHP field officers, who received training beforehand. A subset of PCPs preferred to complete the vignettes before the training sessions at the training site; in such cases, PCPs were seated separately to minimize response contamination.

Following the intervention, WHP field officers conducted follow-up vignettes at PCP clinics after 2–3 months to allow PCPs to familiarize themselves with the AWaRe Handbook and mobile application and integrate the tool into their daily practice. In addition to the vignettes, PCPs completed an endline survey using a post-intervention questionnaire with open-ended questions to gather feedback on the intervention (Supplementary file S2). The baseline and follow-up data collection took an average of 30 to 40 min per provider.

### Data preparation and analysis

Vignette data collected on paper forms were entered into a Microsoft Excel-based system, designed and tested with 5% of the dataset. Data entry was conducted at the clinical condition level rather than the provider level, as most PCPs completed up to four vignettes. Consequently, analyses were based on case numbers rather than PCP numbers. A co-author with a medical background cross-checked and verified antibiotic prescriptions, followed by classification using the AWaRe framework, with a second verification carried out by another co-author. The dataset was then imported into Stata (version 18, StataCorp, USA) for cleaning and analysis.

Descriptive statistics were used to summarize provider characteristics, with categorical variables reported as frequencies and percentages. Changes in antibiotic prescribing knowledge before and after the intervention were assessed using McNemar’s test for paired binary data. Analyses were stratified by provider type (FPs vs. IPs) and clinical condition (acute diarrhea, cellulitis, CAP, and UTI). The primary analysis assessed changes in overall antibiotic prescription and Watch-category antibiotic use. The secondary analysis examined changes in correct antibiotic prescription according to the WHO AWaRe Handbook recommendations. The perceived usefulness of the intervention was evaluated through an endline survey using a post-intervention questionnaire with open-ended questions. Responses to closed-ended questions were summarized as frequencies and percentages. Open-ended responses were reviewed, and selected participants’ comments are presented to illustrate providers’ perception of the intervention.

### Patient and public involvement

Patients and members of the public were not involved in the design, conduct, reporting, or dissemination plans of this research. The study focused on private PCPs and assessed changes in antibiotic prescribing knowledge using clinical vignettes. Given the nature of the intervention and the study population, direct patient or public involvement was not deemed feasible or applicable.

## Results

A total of 145 PCPs (40 FPs and 105 IPs) attended the training and completed both the baseline and follow-up vignettes. The endline survey, which evaluated the usefulness of the intervention using post-intervention questionnaire, was completed by 143 PCPs, as two declined to participate.

### Characteristics of the study population

The median age was 46.5 (24–87) years for FPs and 40 years (18–87) for IPs. Both groups predominantly consisted of male participants, with 98% among FPs and 98% among IPs. The median practice duration was 15 years in both groups. All FPs were either MBBS (72.5%) or Doctor of Medicine (MD) (27.5%) degree holders, while most IPs (80%) had higher education, and a small proportion (6.5%) held degrees in AYUSH (Table 2).

**Table 2:** Distribution of the characteristics of the private primary care providers (PCPs) (n = 145)

Private PCPs demographics		Formal PCPs (n = 40)	Informal PCPs (n = 105)
Age (years) (Median, range)		46.5, 24–87	40, 18–87
Sex n (%)	Male	39 (97.5)	103 (98.1)
	Female	1 (2.5)	2 (1.9)
Service duration (years) (Median, range)		15, 2–45	15, 1–45
Number of patients seen each day (Median, range)		15, 5–100	10, 4–100
Highest level of education achieved n (%)	Primary	0 (0)	3 (2.8)
	Secondary	0 (0)	11 (10.7)
	Higher education*	0 (0)	84 (80)
	MBBS	29 (72.5)	0 (0)
	MD	11 (27.5)	0 (0)
	Bachelor in AYUSH	0 (0)	6 (5.6)
	Master in AYUSH	0 (0)	1 (0.9)

*Higher education among informal PCPs refers to non-health-related tertiary education, such as arts or commerce

### Change in overall antibiotic prescription and Watch-category antibiotic prescriptions for four infectious clinical vignettes before and after the intervention

There were significant changes in antibiotic prescribing following the intervention among acute diarrhea and UTI cases (Table 3). For acute diarrhea cases (n = 145), post-intervention, 23 PCPs stopped prescribing antibiotics while four started prescribing, resulting in a significant net reduction (*p* = 0.0003). Similarly, for UTI cases (n = 144), post-intervention, 31 PCPs stopped prescribing antibiotics while 14 started prescribing antibiotics (*p* = 0.0113). No significant changes were found for cellulitis (*p* = 0.3692) or CAP (*p* = 0.7150).

**Table 3:** Change in overall antibiotic prescription and Watch-category antibiotic prescriptions for four infectious clinical vignettes pre and post intervention among private primary care providers:

Vignette condition	Pre: Prescribed → Post: Prescribed	Pre: Prescribed → Post: Not prescribed	Pre: Not prescribed → Post: Prescribed	Pre: Not prescribed → Post: Not prescribed	p value
*Overall antibiotic prescription*					
Acute diarrhea (n = 145)	79	23	4	39	0.0003*
Cellulitis (n = 145)	91	18	13	23	0.3692
CAP (n = 144)	103	16	14	11	0.7150
UTI (n = 144)	59	31	14	40	0.0113*
*Watch category antibiotic prescription***†**					
Acute diarrhea (n = 145)	38	37	8	62	< 0.0001*
Cellulitis (n = 145)	46	18	28	53	0.1404
CAP (n = 144)	33	29	18	64	0.1086
UTI (n = 144)	43	24	14	63	0.1048

*McNemar’s test (statistically significant at *p* < 0.05)

^†^At least one Watch-category antibiotic prescribed (in case of multiple prescriptions)

In the Watch-category antibiotic prescribing, a significant reduction (*p* < 0.0001) was noted among acute diarrhea cases, with 37 PCPs stopping and eight starting Watch antibiotic use. No significant changes were observed for cellulitis (*p* = 0.1404), CAP (*p* = 0.1086), or UTI (*p* = 0.1048).

### Change in overall antibiotic prescription and antibiotic prescription in concordance with AWaRe Handbook for four clinical vignettes before and after intervention by provider type

Among IPs (n = 105), antibiotic prescribing significantly declined post-intervention for acute diarrhea (*p* = 0.0044) and UTI (*p* = 0.0090), with more providers stopping than starting antibiotic use. No significant changes were observed for cellulitis (*p* = 0.4807) or CAP (*p* = 0.4049). Among FPs (n = 40), a reduction in antibiotic prescribing for acute diarrhea was noted post-intervention but was not statistically significant (*p* = 0.0703), and prescribing patterns remained stable for cellulitis (*p* = 1.0000), CAP (*p* = 0.4531), and UTI (*p* = 1.0000).

Regarding antibiotic prescribing in concordance with the WHO AWaRe Handbook, IPs showed a significant improvement post-intervention for acute diarrhea cases (*p* = 0.0044) and a significant decline for cellulitis cases (*p* = 0.0125). A trend towards improvement was also noted for CAP cases (*p* = 0.0987), although it was not statistically significant. Among FPs, no statistically significant improvements were observed across any condition. However, a trend towards improvement was noted for acute diarrhea cases (*p* = 0.0703) (Table 4).

**Table 4:** Pre and post intervention changes in overall antibiotic prescription and antibiotic prescription in concordance with the WHO AWaRe Antibiotic Handbook among private primary care providers (PCPs), including formal PCPs and informal PCPs in Patna, India

Vignette condition and provider type	Pre: Prescribed → Post: Prescribed	Pre: Prescribed → Post: Not prescribed	Pre: Not prescribed → Post: Prescribed	Pre: Not prescribed → Post: Not prescribed	p-value
*Overall change in antibiotic prescription*					
Acute diarrhea, Formal PCPs (n = 40)	11	7	1	21	0.0703
Acute diarrhea, Informal PCPs (n = 105)	68	16	3	18	0.0044*
Cellulitis, Formal PCPs (n = 40)	21	7	6	6	1.0000
Cellulitis, Informal PCPs (n = 105)	70	11	7	17	0.4807
CAP, Formal PCPs (n = 40)	28	2	5	5	0.4531
CAP, Informal PCPs (n = 104)	75	14	9	6	0.4049
UTI, Formal PCPs (n = 40)	10	6	5	19	1.0000
UTI, Informal PCPs (n = 104)	49	25	9	21	0.0090*

Vignette condition and provider type	Pre: Concordant → Post: Concordant	Pre: Concordant → Post: Not concordant	Pre: Not concordant → Post: Concordant	Pre: Not concordant → Post: Not concordant	p-value
^&^*Antibiotic prescription in concordance with the WHO AWaRe Antibiotic Handbook*					
Acute diarrhea, Formal PCPs (n = 40)	21	1	7	11	0.0703
Acute diarrhea, Informal PCPs (n = 105)	18	3	16	68	0.0044*
Cellulitis, Formal PCPs (n = 40)	6	5	5	24	1.0000
Cellulitis, Informal PCPs (n = 105)	12	21	7	65	0.0125*
CAP, Formal PCPs (n = 40)	12	4	3	21	1.0000
CAP, Informal PCPs (n = 104)	31	10	20	43	0.0987
UTI, Formal PCPs (n = 40)	4	3	3	30	1.0000
UTI, Informal PCPs (n = 104)	3	13	6	82	0.1671

^&^Please refer to Table 1 for the antibiotic prescription definitions in concordance with the WHO AWaRe Handbook

*McNemar’s exact test (statistically significant at *p* < 0.05)

### Participants’ feedback from the endline survey on perceived usefulness of the intervention

The majority of respondents found the intervention useful, with 75% (n = 107) rating the training on the AWaRe Handbook as moderately or slightly useful, including 43.3% (n = 62) who described it as very useful. Similarly, 84.5% (n = 121) found the printed Handbook or infographics useful, with 33.5% (n = 48) rating them as very useful. In contrast, the mobile application received mixed feedback; 26.6% (n = 38) reported that it was not at all useful, 37% (n = 53) found it slightly useful, and only 9.8% (n = 14) rated it as very useful. Additionally, 91.7% (n = 131) of PCPs indicated they would recommend the program to their peers. Table 5 also presents selected illustrative comments from PCPs, drawn from open-ended questions included in the post-intervention questionnaire, to contextualize these findings.

**Table 5:** Summary of private primary care providers (PCPs) responses on the perceived usefulness of the intervention (n = 143)*

Variables	Categories	n (%)	Illustrative participant comments from open-ended responses
Found the training on the WHO AWaRe Antibiotics Handbook useful	Not at all useful	0 (0)	Formal private PCPs (FPs) emphasized how the training enhanced their understanding of responsible antibiotic use following the WHO guidelines, with one noting, *“I learned to prescribe antibiotics as per the WHO guidelines.”* Informal private PCPs (IPs) similarly appreciated the training, as reflected by comments like *“The training taught us to use antibiotics with the correct doses”* and *“I gained a lot of useful knowledge about diseases.”* The practical benefits of the program were further summarised by another IP who shared, *“I learned when and which antibiotics to use.”*
	Slightly useful	16 (11.2)	
	Moderately useful	65 (45.4)	
	Very useful	62 (43.3)	
Found the printed WHO AWaRe Antibiotic Handbook (for formal providers) or infographics (for informal providers) useful	Not at all useful	0 (0)	FPs appreciated the clarity and comprehensiveness of the handbook, with one mentioning, *“It provided detailed information on the correct use of antibiotics”* and another FP emphasising its practical utility, stating, *“The handbook is very useful for selecting antibiotics”*. IPs also highlighted its usefulness, with one noting, *“The handbook helps us understand how to prescribe antibiotics correctly,”* and another IP adding, *“The infographics are easy to understand and very helpful.”* Additionally, the accessibility and ease of reference were valued by IPs, as one stated, *“I use the handbook from time to time for better practice.”* Another IP reinforced its role in improving prescribing, saying, *“The infographics are easy to understand and guide us well.”*
	Slightly useful	22 (15.3)	
	Moderately useful	73 (51)	
	Very useful	48 (33.5)	
Found the WHO AWaRe Antibiotic Handbook mobile application useful	Not at all useful	38 (26.6)	Both FPs and IPs reported no significant usage of the mobile application. Several PCPs noted that they had not downloaded or used the app, indicating potential barriers such as limited familiarity with digital tools
	Slightly useful	53 (37)	
	Moderately useful	38 (26.6)	
	Very useful	14 (9.8)	
Recommend the program to your peers	Yes	131(91.7)	
	No	3 (2)	
	Maybe	9 (6.2)	
Future recommendations	Both FPs and IPs recommended organizing the training periodically, with one suggesting, “*From time to time, organize this type of training,”* and another adding, *“Training should be provided regularly for better practice.”* Another participant emphasized the importance of reaching rural areas, saying, *“Rural practitioners require training on a regular basis.”* To improve engagement, some providers suggested using interactive formats, with one noting, *“It should be explained in a better way through audio-visual aids.”* Additionally, a few IPs highlighted the need for language adaptation, recommending that “*Training modules should also be prepared in Hindi.”*		

*2 PCPs did not participate in the endline survey

## Discussion

Our study provides new insights into the impact of the WHO AWaRe Handbook training in a setting with widespread antibiotic use. The intervention significantly reduced antibiotic prescribing for acute diarrhea and UTI, particularly among IPs. A significant reduction in Watch-category antibiotic use for acute diarrhea was also observed. However, no significant changes were found for cellulitis or CAP, highlighting condition-specific variations in prescribing knowledge.

The lack of change in overall antibiotic prescriptions for cellulitis and CAP cases is expected, as antibiotics are generally indicated for these conditions, and their treatment does not rely on laboratory tests, as outlined in the AWaRe Handbook. A significant decrease in antibiotic prescriptions for UTI cases among IPs is particularly interesting. One possible explanation is that the AWaRe Handbook recommends antibiotic treatment only if there is a compatible clinical presentation and a positive test result (e.g., positive urine leucocytes/leucocyte esterase or positive urine culture). Since our clinical vignettes did not include urine examination or culture results, some providers may not have immediately prescribed antibiotics, contributing to the reduction in antibiotic prescriptions during the post-intervention phase. We did not systematically collect laboratory diagnostic information for the clinical vignettes, so we could not explore whether confirmatory urine tests increased during the post-intervention phase.

The intervention had mixed effects on the prescription of Watch-category antibiotics. A significant reduction in acute diarrhea was observed, but no significant changes were observed in cellulitis, CAP, or UTI. The decrease in Watch-category use for acute diarrhea was primarily driven by reduced overall antibiotic prescribing among IPs. A non-significant declining trend in Watch antibiotic use was observed for CAP, primarily among IPs who shifted from Watch to Access-category antibiotics. For UTI, no significant change in Watch-category prescribing was observed.

The lack of a significant decline in Watch-category use for cellulitis, CAP, and UTI cases highlights the complexity of decision-making regarding antibiotic selection. Several factors may have influenced this trend, including limited familiarity with Access-group antibiotics, lack of awareness of generic names due to reliance on branded medications, and the perception that Access antibiotics are less effective. For instance, a recent study examining the antibiotic stocks of 196 IPs in West Bengal, India, found that none of the IPs stocked nitrofurantoin or trimethoprim-sulfamethoxazole, both of which are WHO-recommended first-line treatments for UTI [34]. For cellulitis, Watch-category antibiotic use remained high (among all other conditions), and there was a significant decline in concordance with the WHO AWaRe Handbook antibiotic prescribing post-intervention among IPs. Cellulitis was intentionally not included in the case-based discussion during the training sessions, unlike the other three conditions. Although cellulitis was included in the education materials provided (Handbook for FPs and an infographic for IPs), the absence of active discussion was intended to allow an exploratory assessment of whether passive provision of guideline-based resources alone could influence prescribing knowledge. While not a main objective of this study, the findings suggest that passive educational material alone may be insufficient to change prescribing knowledge without interactive training. Additionally, acute diarrhea, UTI, and CAP are frequently encountered in primary care settings in India [35], meaning providers likely had prior experience managing these cases and may have felt more confident adjusting their prescribing behaviour. In contrast, cellulitis presents diagnostic uncertainty and perceived severity, leading PCPs, particularly IPs, to favour broad-spectrum antibiotics as a precautionary measure, a pattern reported in previous research [6, 36].

Overall, the observed reductions in antibiotic prescriptions (for some conditions) align with previous research demonstrating the effectiveness of targeted training interventions in improving primary care providers' knowledge and behaviour of appropriate antibiotic use [37]. Similar trends have been reported in studies specifically focused on IPs [38, 39]. These findings highlight the potential of training programs to improve knowledge while demonstrating the need for condition-specific, sustained strategies to modify antibiotic prescribing effectively. While a change in knowledge is essential to influence antibiotic prescribing, the findings also demonstrate that knowledge alone is insufficient to achieve full adherence to the guidelines. Multiple contextual factors shape providers' prescribing behaviour, including the availability of Access-category antibiotics, access to diagnostics, patient demand for antibiotics, economic incentives, provider characteristics, pharmaceutical industry influence, and healthcare system constraints [7, 36, 40]. Training combined with audit-feedback mechanisms, ongoing education, peer engagement, regulatory support and enforcement, and patient or community education campaigns has proven effective among PCPs in various settings [37, 39]. Furthermore, as our intervention was a one-time training delivered to PCPs, sustained interventions with more extended follow-up periods, refresher training and reinforcement sessions are needed for long-term impact [37, 39], which was also one of the key recommendations made by the PCPs who participated in our research.

It is important to note that our study used clinical vignettes to assess changes in prescribing knowledge rather than actual prescribing practices. Vignettes are a well-established tool for evaluating provider decision-making in hypothetical scenarios, but do not fully capture real-world prescribing behaviours, which are influenced by factors such as patient demand, provider characteristics, and healthcare system constraints or incentives [6, 7]. Therefore, our findings should be interpreted as indicative of improvements in knowledge rather than definitive changes in actual clinical practice. Future studies should complement vignette-based assessments with prescription audits, standardized patient methods, or observational data to assess actual prescribing behaviours.

A key observation from this study was the greater impact of training on IPs, who exhibited significant reductions in antibiotic prescribing for some conditions, such as acute diarrhea (16 reduced versus three increased; *p* = 0.0044), and substantial improvements in prescribing concordant with WHO AWaRe Handbook recommendations for acute diarrhea, while FPs showed smaller, non-significant changes. This difference may be explained by lower baseline awareness of antibiotics among IPs, as reported in surveys [41, 42], making them more receptive to training intervention. For instance, a randomized controlled trial conducted in West Bengal, India, demonstrated that IPs showed greater improvements in quality of care following a structured training programme [12].

Our results indicate that all IPs (100%) found the training useful to some extent, ranging from slightly to very useful. This finding is particularly encouraging, as a study from India involving multiple key stakeholders has similarly recommended interventions for IPs that promote optimal guidelines for a limited range of antibiotics and healthcare conditions [34]. Our study also highlights the importance of adapting the AWaRe Handbook to the local context. Specifically, translating infographics into local languages enhanced accessibility for IPs who are less comfortable with English. While printed materials were well received, the digital application was less widely used, possibly due to low digital literacy and providers' apprehension about using a medical application, as reported in other studies [43]. Although a digital application was included in our study, the primary emphasis remained on the printed AWaRe Handbook and infographics. Future research should assess the usability of digital applications, explore alternative formats to enhance accessibility, and incorporate strategies to build digital competencies among providers.

## Strengths and limitations

This study evaluates the impact of training private PCPs on the AWaRe Handbook in India. The use of clinical vignettes allowed for a standardized assessment of prescribing knowledge, and the inclusion of both IPs and FPs provides preliminary, valuable insights into differential training impacts. Additionally, the study was conducted in collaboration with a local NGO, enhancing its real-world applicability and scalability of similar interventions. However, the study also has several limitations. The use of clinical vignettes enables effective measurement of knowledge, but it does not directly reflect the providers' real-world prescribing practices. The sample size was relatively small (especially for FPs), which may have reduced the statistical power to detect moderate effects. Furthermore, the 2–3 months follow-up may not have been sufficient to capture long-term changes in antibiotic prescribing.

## Conclusion

This study provides preliminary evidence on the impact of training private PCPs using the WHO AWaRe Antibiotic Handbook to improve antibiotic prescribing knowledge. The intervention significantly improved prescribing knowledge for acute diarrhea, with a greater impact among IPs, highlighting their responsiveness to structured training. The majority of PCPs rated the training as moderately or very useful. While these findings support the potential of the WHO AWaRe Antibiotic Handbook as an intervention, they also highlight the need for tailored approaches and sustained engagement to achieve long-term improvements in antibiotic prescribing. Future research should focus on longer follow-up periods, larger randomized controlled trials, and real-world prescribing data to evaluate the long-term impact and scalability of such interventions in LMICs' primary care settings.

## Supplementary Information

Below is the link to the electronic supplementary material.

Supplementary Material 1

Supplementary Material 2

## Data Availability

The data underlying the study's results are available in an anonymized format upon reasonable request by emailing the corresponding author at [thapaposhan2009@gmail.com] (mailto:thapaposhan2009@gmail.com).
