# Supplementary material for: Impact of WHO AWaRe Antibiotic Handbook training on antibiotics prescribing knowledge among private primary care providers: a vignette-based, prep–post pilot study in Patna, India
Source: Antimicrob Resist Infect Control. 2026 Mar 27;15:67. doi: 10.1186/s13756-026-01735-6 (PMC13151071; doi:10.1186/s13756-026-01735-6)
Supplement: Supplementary file 1 — Supplementary Material 1 [file 13756_2026_1735_MOESM1_ESM.pdf]

तीव्र संक्रमक दस्त/गैस्ट्रोएंटेराइटिस

वयस्क

परिभाषा

दस्त की नई (<14दिन) शुरुआत (24 घंटे में ⩾3 बेडौल/तरल मल या व्यक्ति के लिए सामान्य से अधिक)। दस्त पानीयुक्त या खूनी हो सकता है (पेचिश)

सर्वाधिक संभावित रोगजनक

पानी जैसा दस्त होना:
- सबसे संभावित कारण वायरल है (अधिकतर रोटावायरस या नोरोवायरस)

खूनी दस्त (पेचिश):
- सबसे संभावित कारण बैक्टीरिया हैं, अधिकतर:
  - –शिगेला एसपीपी
  - –कैम्पिलोबैक्टर एसपीपी
  - – डायरिया गैर–टाइफाइडल साल्मोनेला
  - – एंटरोटॉक्सिजेनिक इशरीकिया कोली

रोकथाम

सुरक्षित पेयजल तक पहुंच, बेहतर स्वच्छता का उपयोग, साबुन से हाथ धोना, अच्छी भोजन स्वच्छता, ये संक्रमण कैसे फैलते हैं इसके बारे में स्वास्थ्य शिक्षा।

Rx इलाज

महत्वपूर्ण:

तीव्र संक्रामक दस्त के लिए पुनर्जलीकरण और इलेक्ट्रोलाइट प्रतिस्थापन मुख्य उपचार है

पर्याप्त मात्रा में तरल पदार्थ पीने से तरल पदार्थ के नुकसान की भरपाई की जा सकती है

दस्तरोधी और वमनरोधी दवाओं की नियमित रूप से आवश्यकता नहीं होती है (वे निर्जलीकरण को नहीं रोकते हैं या पोषण संबंधी स्थित में सुधार नहीं करते हैं)

आमतौर पर एंटीबायोटिक्स की आवश्यकता नहीं होती है, जिसमें गंभीर निर्जलीकरण के मामले भी शामिल हैं

एंटीबायोटिक उपचार पर तभी विचार करें यदि:

–महत्वपूर्ण तीव्र खूनी दस्त

Rx एंटीबायोटिक उपचार

वाच श्रेणी:
एजिथ्रोमाइसिन ओरल
दिन 1: 500 मिलीग्राम q24h
दिन 2–4: 250 मिलीग्राम q24h
उपचार की अवधि: 4 दिन
————————–या–———————
सेफिक्सिम 400 मिलीग्राम q24h (मौखिक)
उपचार की अवधि: 3 दिन
——————————–या–———————————
एक्सेस श्रेणी:
सेफ्टिएक्सोन 1 ग्राम q24h (IV/IM)
उपचार की अवधि: 3दिन

निदान

नैदानिक प्रस्तुति

- दस्त, मतली, उल्टी, सूजन, पेट में दर्द और ऐंठन; बुखार अनुपस्थित हो सकता है
- अधिकांश मामले कुछ ही दिनों में अपने आप ठीक हो जाते हैं
- मरीज अलग–अलग डिग्री के निर्जलीकरण के साथ उपस्थित हो सकते हैं और गंभीर कुपोषण (जोखिम कारक और दस्त का परिणाम दोनों) के साथ उपस्थित हो सकते हैं।

महत्वपूर्ण:
- निर्जलीकरण की डिग्री का तेजी से मूल्यांकन करें (विशेषकर बुजुर्गों में)
- गंभीर निर्जलीकरण के लक्षण (दो या अधिक)। अनिवार्य उपस्थित:
  - –सुस्ती और/या बेहोशी
  - –धंसी हुई आंखें
  - – पीने में असमर्थता
  - – त्वचा की चुभन बहुत धीरे–धीरे वापस जाती है (⩾2 सेकंड)

माइक्रोबायोलॉजी परीक्षण

आमतौर पर जरूरत नहीं होती है।
यदि परीक्षण पर विचार करें: खूनी दस्त
विचारणीय परीक्षण: मल संस्कृति

अन्य प्रयोगशाला परीक्षण

आमतौर पर इसकी जरूरत नहीं होती है लेकिन गंभीर मामलों में विचार करें (उदाहरण के लिए, इलेक्ट्रोलाइट्स की जांच करें)

इमेजिंग

आमतौर पर जरूरत नहीं होती

तीव्र संक्रमक दस्त/गैस्ट्रोएंटेराइटिस

बच्चे

परिभाषा

दस्त की नई (<14दिन) शुरुआत (24 घंटे में ≥3 बेडौल/तरल मल या व्यक्ति के लिए सामान्य से अधिक)। दस्त पानीयुक्त या खूनी हो सकता है (पेचिश)

सर्वाधिक संभावित रोगजनक

पानी जैसा दस्त होना:
- सबसे संभावित कारण वायरल है अधिकतर
  —रोटावायरस
  —नोरोवायरस
  —एडेनोवायरस

खूनी दस्त (पेचिश):
- सबसे संभावित कारण बैक्टीरिया हैं, अधिकतर:
  —शिगेला एसपीपी
  —कैम्पिलोबैक्टर एसपीपी
  — डायरिया गैर—टाइफाइडल साल्मोनेला
  — एंटरोटॉक्सिजेनिक इशरीकिया कोली

रोकथाम

- सुरक्षित पेयजल तक पहुंच, बेहतर स्वच्छता का उपयोग, साबुन से हाथ धोना, अच्छी भोजन स्वच्छता, ये संक्रमण कैसे फैलते है इसके बारे में स्वास्थ्य शिक्षा
- रोटावायरस के विरूद्ध टीकाकरण

निदान

नैदानिक प्रस्तुति

- दस्त, मतली, उल्टी, सूजन, पेट में दर्द और ऐंठन
- अधिकांश मामले कुछ ही दिनों में अपने आप ठीक हो जाते हैं

महत्वपूर्ण
- निर्जलीकरण की डिग्री का तेजी से मूल्यांकन करें
- गंभीर निर्जलीकरण के लक्षण (दो या) और अधिक मौजूद होना चाहिए:
  —सुस्ती और/या बेहोशी
  —धंसी हुई आंखें
  —पीने में असमर्थता
  —त्वचा की चुभन बहुत धीरे—धीरे वापस जाती है (≥2सेकंड)

माइक्रोबायोलॉजी परीक्षण

आमतौर पर जरूरत नहीं होती है।
यदि परीक्षण पर विचार करें: खूनी दस्त
विचारणीय परीक्षण: मल संस्कृति

अन्य प्रयोगशाला परीक्षण

आमतौर पर इसकी जरूरत नहीं होती है लेकिन गंभीर मामलों में विचार करें (उदाहरण के लिए, इलेक्ट्रोलाइट्स की जांच करें)

इमेजिंग

आमतौर पर जरूरत नहीं होती

Rx इलाज

कोई एंटीबायोटिक देखभाल नहीं

महत्वपूर्ण: तीव्र संक्रामक दस्त के लिए पुनर्जलीकरण और इलेक्ट्रोलाइट प्रतिस्थापन मुख्य उपचार है।
- कम ऑस्मोलैरिटी ओरल डिहाईड्रेशन सॉल्यूशन (ओआरएस) की सिफारिश की जाती है।
- ओआरएस के अलावा, 10–14 दिनों के लिए जिंक की गोलियां (10–20 मिलीग्राम/दिन) लक्षणों की अवधि और गंभीरता को कम कर सकती है।

दस्तरोधी और वमनरोधी दवाओं की नियमित रूप से आवश्यकता नहीं होती है (वे निर्जलीकरण को नहीं रोकते हैं या पोषण संबंधी स्थिति में सुधार नहीं करते हैं)

नैदानिक विचार:
- आमतौर पर एंटीबायोटिक दवाओं की आवश्यकता नहीं होती है, जिसमें बुखार और/या गंभीर निर्जलीकरण के मामले भी शामिल है।
- एंटीबायोटिक उपचार पर केवल तभी विचार करें यदि:
  —महत्वपूर्ण खूनी दस्त

एंटीबायोटिक उपचार

वाच श्रेणी:
एजिथ्रोमाइसिन 10 मिलीग्राम/कि.ग्रा/खुराक q24h (ओरल)
उपचार की अवधि: 4 दिन
————————या——————————
सेफिक्सिम 10 मिलीग्राम/कि.ग्रा/खुराक q24h (ओरल)
उपचार की अवधि: 5 दिन
——————————————या——————————————

एक्सेस श्रेणी:
सेफ्टिएक्सोन 80 मिलीग्राम/कि.ग्रा/खुराक q24h (IV/IM)
उपचार की अवधि: 3दिन

स्त्रोत: WHO AWaRe (एक्सेस, वॉच, रिजर्व) से अनुकूलित ई) एंटीबायोटिक पुस्तक

निचले मूत्र पथ का संक्रमण

वयस्क

परिभाषा

• मूत्र पथ के निचले हिस्से का संक्रमण (जैसे, मूत्राशय– सिस्टिटिस)
• मूत्र पथ की संरचनात्मक विसंगतियों वाले या जिनकी प्रतिरक्षा प्रणाली कमजोर हैं और गर्भवती महिलाओं में मूत्र पथ संक्रमण (यूटीआई) को आमतौर पर जटिल विकास (जटिल यूटीआई) के अधिक जोखिम में माना जाता है।

सर्वाधिक संभावित रोगजनक

जीवाणु:
• अत्यंत साधारण
–एंटरोबैक्टेरेलिस (ज्यादातर इशरीकिया कोली जिसमें ईएसबीएल एंजाइम का उत्पादन करने वाले मल्टीड्रग प्रतिरोधी शामिल है)

निदान

नैदानिक प्रस्तुति

तीव्र (<1 सप्ताह) डिसुरिया, मूत्र की तात्कालिकता और आवृत्ति में वृधि, पेट के निचले हिस्से में दर्द या बेचैनी और कभी–कभी अत्यधिक रक्तमेह

महिलाओं में, योनि स्त्रोत से संबंधित (योनि स्त्राव या जलन) को पहले पहचान करके रखा जाना चाहिए।

माइक्रोबायोलॉजी परीक्षण

रोगसूचक रोगियों में यदि बार–बार यूटीआई हो तो यूरिन कल्चर

अन्य प्रयोगशाला परीक्षण

आमतौर पर जरूरत नहीं होती

इमेजिंग

आमतौर पर जरूरत नहीं होती

इलाज

नैदानिक विचार

यदि नैदानिक प्रस्तुति संगत हो तो एंटीबायोटिक उपचार की सिफारिश की जाती है।

उपचार की अवधि उपयोग किए गए एंटीबायोटिक के अनुसार भिन्न होती है – संबंधित एंटीबायोटिक अनुभाग देखें

टिप्पणी: सामान्य तौर पर गर्भवती महिलाओं (आमतौर पर 5 दिन) और पुरूषों (आमतौर पर 7 दिन) के लंबे उपचार पर विचार करें।

एंटीबायोटिक उपचार

नाइट्रोफ्यूरेटोइन (ओरल)
100 मिलीग्राम q12h (संशोधित रिलीज फॉरमूलेशन)
उपचार की अवधि: 5 दिन

————————या————————

एमोक्सिसिलिन + क्लेवुलेनीक एसिड
500 मिलीग्राम + 125 मिलीग्राम q8h (ओरल)
उपचार की अवधि: 5 दिन

————————या————————

सल्फामेथोक्साजोल + ट्राइमेथोप्रिम
500 मिलीग्राम + 160 मिलीग्राम q12h (ओरल)
उपचार की अवधि: 3 दिन

निचले मूत्र पथ का संक्रमण

बच्चे

परिभाषा

• मूत्र पथ के निचले हिस्से का संक्रमण (जैसे, मूत्राशय–सिस्टिटिस)
• मूत्र पथ की संरचनात्मक विसंगतियों (उदाहरण के लिए, वेसिकोयूरेटरल रिफ्लक्स या अन्य जन्मजात विसंगतियों) वाले या जिनकी प्रतिरक्षा प्रणाली कमजोर होती हैं उनमें मूत्र पथ संक्रमण (यूटीआई) को आमतौर पर इसके अधिक जोखिम में माना जाता है। जटिल विकास (जटिल यूटीआई)

सर्वाधिक संभावित रोगजनक

जीवाणु:
• **अत्यंत साधारण:**
–एंटरोबैक्टेरेलिस (ज्यादातर *इशरीकिया कोली* जिसमें ईएसबीएल एंजाइम का उत्पादन करने वाले मल्टीड्रग प्रतिरोधी उपभेद शामिल है)

निदान

नैदानिक प्रस्तुति

- तीव्र (<1 सप्ताह) डिसुरिया, मूत्र की तात्कालिकता और आवृत्ति में वृद्धि, असंयम/गीलापन, पेट के निचले हिस्से में दर्द, या बेचैनी और कभी–कभी अत्यधिक रक्तमेह
- आमतौर पर, कोई प्रणालीगत लक्षण/लक्षण नहीं (उदाहरण के लिए बुखार)
- लड़कियों में, लक्षणों के योनि स्त्रोत (योनि स्त्राव या जलन) को पहले पहचान करके रखा जाना चाहिए

माइक्रोबायोलॉजी परीक्षण

रोगसूचक रोगियों में: निदान की पुष्टि करने और अनुभवजन्य उपचार को अनुकूलित करने के लिए यूरिन कल्चर (हमेशा बच्चों में)

अन्य प्रयोगशाला परीक्षण

आमतौर पर जरूरत नहीं होती

इमेजिंग

आमतौर पर इसकी आवश्यकता नहीं होती जब तक कि मूत्र पथ की संभावित अंतःनिहित असामान्यताओं की जांच करने की आवश्यकता न हो

इलाज

नैदानिक विचार

अनुकूल नैदानिक प्रस्तुति और सकारात्मक परीक्षण होने पर एंटीबायोटिक उपचार की सिफारिश की जाती है (सकारात्मक मूत्र ल्यूकोसाइट्स/ल्यूकोसाइट एस्टरेज या सकारात्मक यूरिन कल्चर)
- यदि परीक्षण नहीं किया जा सका, तो नैदानिक प्रस्तुति के आधार पर उपचार करें।
- नैदानिक सुधार 48–72 घंटों के भीतर स्पष्ट होना चाहिए
- एंटीबायोटिक्स लक्षणों की अवधि 2 दिन कम कर देते है

टिप्पणी: उपचार की अवधि उपयोग किए गए एंटीबायोटिक के अनुसार भिन्न होती है – संबंधित एंटीबायोटिक अनुभाग देखें

एंटीबायोटिक उपचार

ACCESS
नाइट्रोफ्यूरटाइन (ओरल)
100 मिलीग्राम q12h (संशोधित रिलीज फॉरमूलेशन)
उपचार की अवधि: 5 दिन

——————————या——————————

ACCESS
एमोक्सिसिलिन + क्लैवुलैनीक एसिड
80–90 मिलीग्राम/कि.ग्रा./दिन एमोक्सिसिलिन घटक (ओरल)

ओरल वजन बैंड:

-<6 किग्रा	250 मिलीग्राम एमोक्स/खुराक q12h
-<10 किग्रा	375 मिलीग्राम एमोक्स/खुराक q12h
0-<15 किग्रा	500 मिलीग्राम एमोक्स/खुराक q12h
5-<20 किग्रा	750 मिलीग्राम एमोक्स/खुराक q12h
20 किग्रा	500 मिलीग्राम एमोक्स/खुराक q8h<br>या 1 ग्राम एमोक्स/खुराक q12h

उपचार की अवधि: 3–5 दिन

——————————या——————————

ACCESS
सल्फामेथोक्साजोल + ट्रामेथोप्रिम
20 मिलीग्राम/कि.ग्रा. q12h (ओरल)

ओरल वजन बैंड:

-<6 किग्रा	100 मिलीग्राम + 20 मिलीग्राम q12h
-<10 किग्रा	200 मिलीग्राम+ 40 मिलीग्राम q12h
0-<30 किग्रा	400 मिलीग्राम+ 80 मिलीग्राम q12h
30 किग्रा	800 मिलीग्राम+ 160 मिलीग्राम q12h

उपचार की अवधि: 3–5 दिन

स्त्रोत: WHO AWaRe (एक्सेस, वॉच, रिजर्व) से अनुकूलित ई) एंटीबायोटिक पुस्तक

कोशिका शोथ

वयस्क

परिभाषा

सतही जीवाणु त्वचा संक्रमण, गहरी ऊतक परतों को प्रभावित नहीं करता

निदान

नैदानिक प्रस्तुति

कोशिका: त्वचा पर घाव की तीव्र शुरूआत, जिसमें प्रभावित क्षेत्र में लालिमा, सूजन और सख्तता, गर्मी और दर्द या कोमलता होती है।

- सबसे अधिक प्रभावित क्षेत्र: पैर और चेहरा
- बुखार (≥100.4 डिग्री फारेनहाइट) और प्रणालीगत संक्रमण के अन्य लक्षण मौजूद हो सकते हैं।
- अकेले लालिमा किसी संक्रमण का संकेत नहीं दे सकती

कीटाणु विज्ञान

अधिकांश इनके मामलों में इसकी आवश्यकता नहीं होती

अन्य प्रयोगशाला परीक्षण

अधिकांश इनके मामलों में इसकी आवश्यकता नहीं होती

इमेजिंग

आमतौर पर जरूरी नहीं है

सर्वाधिक संभावित रोगजनक

बैक्टीरिया (ज्यादातर मामले):

- *स्टरेप्टोकोकस प्योगेनेस* (समूह अ स्ट्रैपटोकोकस)
- *स्टाफीलोकोकस ऑरीअस*

इलाज

नैदानिक विचार

- **अनुभवजन्य एंटीबायोटिक विकल्प** अच्छा होना जरूरी है दोनों के खिलाफ कार्रवाई सट्रेप्टोकोकस प्योगेनेस (समूह अ स्ट्रैपटोकोकस) और स्टाफीलोकोकस ऑरीअस
- **हल्का संक्रमण:** मौखिक उपचार पर्याप्त है
- **अंतःशिरा एंटीबायोटिक्स:** यदि संक्रमण तेजी से फैल रहा है और मौखिक एंटीबायोटिक दवाओं का असर नहीं हो रहा है तो इसकी आवश्यकता हो सकती है।

एंटीबायोटिक उपचार की अवधि

5 दिन के इलाज करें

सामयिक उपचार

आवश्यकता नहीं

एंटीबायोटिक उपचार

ACCESS सेफालाक्सीन 500 मिलीग्राम q8h (ओरल)

———————या———————

ACCESS क्लोक्सासीलीन 500 मिलीग्राम q6h (ओरल)

———————या———————

ACCESS अमोक्सीसिलीन + क्लैवुलैनीक एसिड 500 मिलीग्राम + 125 मिलीग्राम q8h (ओरल)

कोशिका शोथ

बच्चे

परिभाषा

सतही जीवाणु त्वचा संक्रमण, गहरी ऊतक परतों को प्रभावित नहीं करता

निदान

नैदानिक प्रस्तुति

कोशिका शोथ: त्वचा पर घाव की तीव्र शुरूआत, जिसमें प्रभावित क्षेत्र में लालिमा, सूजन और सख्तता, गर्मी और दर्द या दर्द के प्रति संवेदनशीलता होती है।

- सबसे अधिक प्रभावित क्षेत्र: पैर और चेहरा
- बुखार (≥100.4 डिग्री फारेनहाइट) और प्रणालीगत संक्रमण के अन्य लक्षण मौजूद हो सकते हैं।
- अकेले लालिमा किसी संक्रमण का संकेत नहीं दे सकती

कीटाणु विज्ञान

अधिकांश इनके मामलों में इसकी आवश्यकता नहीं होती

अन्य प्रयोगशाला परीक्षण

अधिकांश इनके मामलों में इसकी आवश्यकता नहीं होती

इमेजिंग

आमतौर पर जरूरी नहीं है

सर्वाधिक संभावित रोगजनक

बैक्टीरिया (ज्यादातर मामले):

- *स्टरेप्टोकोकस प्योगेनेस* (समूह अ स्ट्रैपटोकोकस)
- *स्टाफीलोकोकस ऑरीअस*

इलाज

नैदानिक विचार

- **अनुभवजन्य एंटीबायोटिक विकल्प** अच्छा होना जरूरी है दोनों के खिलाफ कार्रवाई सट्रेप्टोकोकस प्योगेनेस (समूह अ स्ट्रैपटोकोकस) और स्टाफीलोकोकस ऑरीअस
- **हल्का संक्रमण:** मौखिक उपचार पर्याप्त है
- **अंतःशिरा एंटीबायोटिक्स:** यदि संक्रमण तेजी से फैल रहा है और मौखिक एंटीबायोटिक दवाओं का असर नहीं हो रहा है तो इसकी आवश्यकता हो सकती है।

एंटीबायोटिक उपचार की अवधि

5 दिन के इलाज करें

सामयिक उपचार

आवश्यकता नहीं

एंटीबायोटिक उपचार

ACCESS सेफैलेक्सिन 25 मिलीग्राम / किग्रा / खुराक q12h ओरल

मौखिक वजन बैंड:

3 -<6 किग्रा	125 मिलीग्राम q12h
6 -<10 किग्रा	250 मिलीग्राम q12h
10-<15 किग्रा	375 मिलीग्राम q12h
15-<20 किग्रा	500 मिलीग्राम q12h
20-<30 किग्रा	625 मिलीग्राम q8h
30 किग्रा	500 मिलीग्राम q8h

ACCESS क्लोक्सालीन 15 मिलीग्राम / किग्रा / खुराक q6h ओरल

मौखिक वजन बैंड:

3 -<6 किग्रा	62.5 मिलीग्राम q6h
6 -<10 किग्रा	125 मिलीग्राम q6h
10-<15 किग्रा	250 मिलीग्राम q6h
15-<20 किग्रा	375 मिलीग्राम q6h
20 किग्रा	500 मिलीग्राम q6h

ACCESS एमोक्सिसिलीन + ट्रामेथोप्रिम
90 मिलीग्राम / कि.ग्रा. / दिन एमोक्सिलीन घटक (ओरल)

मौखिक वजन बैंड:

-<6 किग्रा	250 मिलीग्राम एमोक्स / खुराक q12h
-<10 किग्रा	375 मिलीग्राम एमोक्स / खुराक q12h
0-<15 किग्रा	500 मिलीग्राम एमोक्स / खुराक q12h
5-<20 किग्रा	750 मिलीग्राम एमोक्स / खुराक q12h
20 किग्रा	500 मिलीग्राम एमोक्स / खुराक q8h<br>या 1 ग्राम एमोक्स / खुराक q12h

स्त्रोत: WHO AWaRe (एक्सेस, वॉच, रिजर्व) से अनुकूलित ई) एंटीबायोटिक पुस्तक

समुदाय उपार्जित निमोनिया

वयस्क

परिभाषा

फेफड़ों को प्रभावित करने वाली एक गंभीर बिमारी जिसमें आमतौर पर खांसी, बलगम आना और सांस लेने में तेज और कठिनाई के साथ छाती के रेडियोग्राफ पर नई या बिगड़ती फुफ्फुसीय घुसपैठ शामिल होती है

सर्वाधिक संभावित रोगजनक

'विशिष्ट' बैक्टीरिया:

- **स्ट्रैपटोकोकस निमोनिया** (अधिकांश मामले)
- *हेमोफिलस इन्फ्लुएंज* (पुरानी फेफड़ों की बीमारियाँ, धुम्रपान)
- *मोराक्सेला कैटरलिस* (पुरानी फेफड़ों की बीमारियाँ, धुम्रपान)

'एटिपिकल' बैक्टीरिया:

- *माइकोप्लाज्मा निमोनिया* (युवा वयस्कों में अधिक बार)
- *क्लैमाइडिया निमोनिया और सिटासी* (युवा वयस्कों में अधिक बार)

श्वसन विषाणु:

- *इन्फ्लूएंजा* विषाणु (ए और बी)
- कोरोना वायरस (SARS-CoV-2 सहित)
- ऐडिनोवायरस

निदान

नैदानिक प्रस्तुति

- नई शुरूआत (<2 सप्ताह) या बुखार के साथ बिगड़ती खांसी (100.4 डिग्री फारेनहाइट), थूक का उत्पादन, सांस की तकलीफ, तचीपनिया, ऑक्सीजन संतृप्ति में कमी, फेफड़े के गुदाभ्रंश पर क्रेपिटेशन, वैकल्पिक स्पष्टीकरण के बिना सीने में दर्द/असुविधा
- बुजुर्गों में एक्स्ट्रापल्मोनरी विशेषताएं (यानी भ्रम, भटकाव) प्रमुख हो सकती है

माइक्रोबायोलॉजी परीक्षण

हल्के मामले: आमतौर पर जरूरत नहीं होती
गंभीर मामले (रोगाणुरोधी उपचार का मार्गदर्शन करने के लिए): रक्त संस्कृतियाँ

अन्य प्रयोगशाला परीक्षण

रोग की गंभीरता का निर्धारण करें: रक्त यूरिया नाइट्रोजन, श्वेत रक्त कोशिका गिनती

इमेजिंग

हल्के मामलों में छाती का एक्स–रे आवश्यक नहीं है

CURB-65 गंभीरता स्कोरिंग प्रणाली

संकेत एवं लक्षण (प्रत्येक 1 अंक)

- भ्रम की उपस्थिति
- यूरिया >19mg/dL (या >7mmol/L)*
- श्वसन दर >30/मिनट
- सिस्टोनिक बीपी <90mmHg (<12केपीए) या डायस्टोलिक बीपी ≤60mmHg (<8केपीए)
- आयु ≥65 वर्ष

स्कोर 0–1
- बाह्य रोगी उपचार पर विचार करें

स्कोर 2
- अस्पताल में इलाज पर विचार करें

स्कोर ≥3
- अस्पताल आईसीयू उपचार

***द सीआरबी–65** स्कोर, जिनकी गणना के लिए प्रयोगशाला मूल्यों की आवश्यकता हीं होती है, का भी उपयोग किया जा सकता है, स्कोर मूल्य व्याख्या CURB-65 के समान है।

इलाज

हल्के से मध्यम मामले:

अमोक्सिसिलिन 1 ग्राम q8h ओरल

————————या————————

फेनोक्सिमिथाइलपेनिसिलिन (पोटेशियम के रूप में) 500 ग्राम (8,00,000 आईयू) q8h ओरल

————————या————————

एमोक्सिसिलन + क्लैवलुलैनीक एसिड 875 मिलीग्राम + 125 मिलीग्राम q8h ओरल

————————या————————

उपचार की अवधि: 5 दिनों में उपचार करें

गंभीर मामले: अस्पताल रेफर करें

समुदाय उपार्जित निमोनिया

बच्चे

परिभाषा

फेफड़ों को प्रभावित करने वाली एक गंभीर बिमारी जिसमें आमतौर पर खांसी होती है और छाती के रेडियोग्राफ पर नई या बिगड़ती फुफ्फुसीय घुसपैठ के साथ तेजी से और सांस लेने में कठिनाई होती है

सर्वाधिक संभावित रोगजनक

'विशिष्ट' बैक्टीरिया:
- **स्ट्रैपटोकोकस निमोनिया** (जीवन के पहले सप्ताह के बाद सीएपी का सबसे आम कारण)
- *हेमोफिलस इन्फ्लुएंज*
- *मोराक्सेला कैटरलिस*

'असामान्य' रोगजनक (छोटे बच्चों की तुलना में 5 वर्ष से अधिक उम्र के बच्चों में अधिक बार):
- *माइकोप्लाज्मा निमोनिया*
- *क्लैमाइडोफिला निमोनिया*

श्वसन विषाणु:
- रेस्पिरेटरी सिंकाइटयल वायरस (आरएसवी)
- *इन्फ्लूएंजा* विषाणु (ए और बी)
- कोरोना वायरस (SARS-CoV-2 सहित)
- ऐडिनोवायरस

निदान

नैदानिक प्रस्तुति

- नई शुरूआत (<2 सप्ताह) या बुखार के साथ बिगड़ती खांसी (≥100.4 डिग्री फारेनहाइट), सांस की तकलीफ, तचीपनिया, कम ऑक्सीजन संतृप्ति, घबराहट, सायनोसिस, घुरघुराहट, नाक का फड़कना, पीलापन
- निमोनिया का निदान उम्र के हिसाब से तेज सांस लेने और/या छाती में खिंचाव होने पर होता है
- यदि उपलब्ध हो तो ऑक्सीजन सैचुरोमीटर से हाइपोक्सिया की जांच करें

माइक्रोबायोलॉजी परीक्षण

हल्के मामले: आमतौर पर जरूरत नहीं होती
गंभीर मामले (रोगाणुरोधी उपचार का मार्गदर्शन करने के लिए): रक्त संस्कृतियाँ

अन्य प्रयोगशाला परीक्षण

विचार करना: पूर्ण रक्त गणना

इमेजिंग

हल्के मामलों में छाती का एक्स–रे आवश्यक नहीं है

℞ इलाज

गंभीरता आकलन और विचार:
बच्चों के साथ **न्यूमोनिया**
- घरेलू देखभाल की सलाह के साथ घर पर ओरल एमोक्सिसिलिन से इलाज किया जाना चाहिए
- निमोनिया का निदान या तो किया जाता है:
  –तेजी से सांस लेना (2–11 महीने की उम्र के बच्चों में श्वसन दर < 50 सांस/मिनट; 1–5 साल की उम्र के बच्चों में श्वसन दर >40 सांस/मिनट)
  – छाती खींचना

बच्चों के साथ **गंभीर निमोनिया** (या निमोनिया से पीड़ित बच्चा जो मौखिक एंटीबायोटिक बर्दाश्त नहीं कर सकता):
- अस्पताल में भर्ती कराया जाना चाहिए और अंत:शिरा एंटीबायोटिक दवाओं से इलाज किया जाना चाहिए
- गंभीर निमोनिया की पहचान निमोनिया के लक्षणों से होती है:
  –तेजी से सांस लेना (+/– छाती को अंदर खींचना) पल्स
  –एक सामान्य खतरे का संकेत:
    - स्तनपान कराने या पीने में असमर्थता
    - आक्षेप
    - सुस्ती या चेतना का कम स्तर

इलाज:
हल्के से मध्यम मामले: अमोक्सिसिलिन 80–90 मिलीग्राम/किग्रा/दिन (ओरल)
ओरल मौखिक बैंड:

3-<6 किग्रा	250 मिलीग्राम q12h
6-<10 किग्रा	375 मिलीग्राम q12h
10-<15 किग्रा	500 मिलीग्राम q12h
15-<20 किग्रा	750 मिलीग्राम q12h
≥ 20 किग्रा	500 मिलीग्राम q8h<br>या 1 ग्रा q12h

उपचार की अवधि:
- 3 दिन: यदि बच्चे की छाती में कोई खिंचाव नहीं है
- 5 दिन: यदि बच्चे की छाती में जलन हो

गंभीर मामले: अस्पताल रेफर करें

ब्रोंकाइटिस

वयस्क

परिभाषा

लगातार खांसी +/- बुखार (≥100.4 डिग्री फारेनहाइट) द्वारा विशेषता श्वासनली और ब्रांकाई की एक सर्व–सीमित सूजन

निदान

नैदानिक प्रस्तुति

• खांसी की तीव्र शुरूआत (<2 सप्ताह) जो 5 दिनों से अधिक समय तक चलती है, +/- थूक का उत्पादन और सांस की तकलीफ (थूक का रंग जीवाणु संक्रमण का संकेत नहीं देता है), +/-बुखार ((≥100.4°F)

• आमतौर पर, हल्की स्थिति, खांसी आमतौर पर 10–20 दिनों तक रहती है (अधिक समय तक भी रह सकती है)

महत्वपूर्ण: लक्षण निमोनिया के साथ ओवरलैप हो सकते हैं, और इससे एंटीबायोटिक दवाओं के साथ अनुचित उपचार हो सकता है। रोगी का सावधानीपूर्वक मूल्यांकन करके इससे बचना चाहिए।

• **ब्रोकाइटिस:** कम गंभीर प्रस्तुति, आमतौर पर स्व–सीमित (लेकिन खांसी को ठीक होने में कई सप्ताह लग सकते हैं)

• **निमोनिया (देखें ''समुदाय–अभिग्रहित निमोनिया'' इन्फोग्राफिक):** सांस की तकलीफ के साथ अधिक गंभीर प्रस्तुति और संक्रमण के प्रणालीगत लक्षण (उदाहरण के लिए हृदय और श्वसन दर में वृद्धि)

माइक्रोबायोलॉजी परीक्षण

आमतौर पर जरूरत नहीं होती

अन्य प्रयोगशाला परीक्षण

आमतौर पर जरूरत नहीं होती

इमेजिंग

आमतौर पर जरूरत नहीं होती

सर्वाधिक संभावित रोगजनक

श्वसन विषाणु:
• रेनो वायरस
• *इन्फ्लूएंजा* विषाणु (ए और बी)
• कोरोना वायरस (SARS-CoV-2 सहित)
• रेस्पिरेटरी सिंकाइटियल वायरस (आरएसबी)

Rx इलाज

कोई एंटीबायोटिक देखभाल नहीं

• लक्षणात्मक इलाज
• ब्रोकोडाईलेटरस (घरघराहट के मामले में), म्यूकोलाईटिक या एंटीट्यूसिव एजेंटों पर स्थानीय प्रथाओं और रोगी की प्राथमिकताओं के आधार पर विचार किया जा सकता है।

मरीजों को सूचित किया जाना चाहिए:
• अधिकांश मामले स्व–सीमित और वायरल मूल के होते हैं
• खांसी कई हफ्तों तक बनी रह सकती है

Rx लक्षणात्मक इलाज

दवाओं को वर्णानुक्रम में सूचीबद्ध किया गया है और उन्हें उपचार के समान विकल्पों पर विचार किया जाना चाहिए

आईब्रुप्रोफेन 200–400 मिलीग्राम क्यू6–8एच (मैक्स. 2.4 ग्रा/दिन

————————————या————————————

पैरासिटामोल (एसिटामिनोफेन) 500 मिलीग्राम–1 ग्राम क्यू4–6 एच (अधिकतम 4 ग्राम/दिन)
• **यकृत हानि/सिरोसिस:** अधिकतम 2 ग्राम/दिन

Rx एंटीबायोटिक इलाज

एंटीबायोटिक उपचार है **अनुशंसित नहीं है और इससे बचना चाहिए** क्योंकि किसी महत्वपूर्ण नैदानिक लाभ का कोई सबूत नहीं है और एंटीबायोटिक दवाओं के दुष्प्रभाव का खतरा है

ब्रोंकाइटिस

बच्चे

परिभाषा

लगातार खांसी +/- बुखार (≥100.4 डिग्री फारेनहाइट) द्वारा विशेषता श्वासनली और ब्रांकाई की एक सर्व–सीमित सूजन

निदान

नैदानिक प्रस्तुति

- खांसी की तीव्र शुरूआत जो 5 दिनों से अधिक समय तक रहती है, आमतौर पर नाक बहने और हल्के बुखार के साथ
- आमतौर पर, हल्की स्थिति वाली खांसी आमतौर पर 1–3 सप्ताह तक रहती है

महत्वपूर्ण: लक्षण निमोनिया के साथ ओवरलैप हो सकते हैं, और इससे एंटीबायोटिक दवाआं के साथ अनुचित उपचार हो सकता है। रोगी का सावधानीपूर्वक मूल्यांकन करके इससे बचना चाहिए।

- **ब्रोंकाइटिस**: कम गंभीर प्रस्तुति, आमतौर पर स्व–सीमित (लेकिन खांसी को ठीक होने में कई सप्ताह लग सकते हैं)
- **निमोनिया (देखें "समुदाय–अभिग्रहित निमोनिया" इन्फोग्राफिक)**: सांस की तकलीफ और संक्रमण के प्रणालीगत लक्षण के साथ अधिक गंभीर प्रस्तुति (उदाहरण के लिए हृदय और श्वसन दर में वृद्धि)

माइक्रोबायोलॉजी परीक्षण

आमतौर पर जरूरत नहीं होती; इन्फ्लूएंजा वायरस या SARS-CoV-2 के परीक्षण पर विचार करें (उदाहरण के लिए, इन्फ्लूएंजा के मौसम के दौरान या स्थानीय स्तर पर फैलने के दौरान महामारी विज्ञान जोखिम/स्थिति /प्रोटोकॉल

अन्य प्रयोगशाला परीक्षण

आमतौर पर जरूरत नहीं होती

इमेजिंग

आमतौर पर जरूरत नहीं होती

सर्वाधिक संभावित रोगजनक

श्वसन विषाणु:
- रेनो वायरस
- *इन्फ्लूएंजा* वायरस (ए और बी)
- पैराइन्फ्लूएंजा वायरस
- कोरोना वायरस (SARS-CoV-2 सहित)
- रेस्पिरेटरी सिंकाइटियल वायरस (आरएसबी)
- मेटान्यूमो वायरस
- ऐडिनो वायरस
- अन्य श्वसन वायरस

इलाज

कोई एंटीबायोटिक देखभाल नहीं

- लक्षणात्मक इलाज
- ब्रोकोडाईलेटरस (घरघराहट के मामले में), म्यूकोलाईटिक या एंटीट्यूसिव एजेंटों पर स्थानीय प्रथाओं और रोगी की प्राथमिकताओं के आधार पर विचार किया जा सकता है।

मरीजों/अभिभावकों को सूचित किया जाना चाहिए कि:
- अधिकांश मामले स्व–सीमित और वायरल मूल के होते हैं
- खांसी कई हफ्तों तक बनी रह सकती है

लक्षणात्मक इलाज

दवाओं को वर्णानुक्रम में सूचीबद्ध किया गया है और उन्हें उपचार के समान विकल्पों पर विचार किया जाना चाहिए

आईब्रुप्रोफेन (3 महीने से कम उम्र होने पर इसका उपयोग न करें)
- दर्द नियंत्रण/ज्वरनाशक : 5–10 मिलीग्राम/ किग्रा क्यू26–7एच
- **ओरल वजन बैंड**

6-<10 किग्रा	50 मिलीग्राम q8h
10-<15 किग्रा	100 मिलीग्राम q8h
15-<20 किग्रा	150 मिलीग्राम q8h
20-<30 किग्रा	200 मिलीग्राम q8h
≥ 30 किग्रा	200–400 मिलीग्राम q6h (अधिकतम 2.4 ग्रा/दिन)

———————————या———————————

पैरासिटामोल (एसिटामिनोफेन)
- दर्द नियंत्रण/ज्वरनाशक : 10–15 मिलीग्राम/किग्रा क्यू6एच
- **ओरल वजन बैंड**

3-<6 किग्रा	60 मिलीग्राम q6h
6-<10 किग्रा	100 मिलीग्राम q6h
10-<15 किग्रा	150 मिलीग्राम q6h
15-<20 किग्रा	200 मिलीग्राम q6h
20-<30 किग्रा	300 मिलीग्राम q6h
≥ 30 किग्रा	500 मिलीग्राम 1 ग्राम q6h (अधिकतम 4 ग्रा/दिन या 2 ग्राम /दिन यदि यकृत संबंधी हानि/सिरोसिस)

एंटीबायोटिक उपचार

एंटीबायोटिक उपचार है **अनुशंसित नहीं है और इससे बचना चाहिए** क्योंकि किसी महत्वपूर्ण नैदानिक लाभ का कोई सबूत नहीं है और एंटीबायोटिक दवाओं के दुष्प्रभाव का खतरा है

स्त्रोतः WHO AWaRe (एक्सेस, वॉच, रिजर्व) से अनुकूलित ई) एंटीबायोटिक पुस्तक

तीव्र साइनस

वयस्क

परिभाषा

पेरानेसल साइनस और नाक गुहा की एक लक्षणात्मक सूजन

सर्वाधिक संभावित रोगजनक

श्वसन विषाणु:
- इन्फ्लूएंजा विषाणु (ए और बी)
- रेनो वायरस
- कोरोना वायरस (SARS-CoV-2 सहित)
- रेस्पिरेटरी सिंकाइटियल वायरस (आरएसबी)

बैक्टीरिया (शायद ही कभी)
- स्ट्रेपटोकोकक्स न्यूमोनिया
- हमोफिलिअस इन्फ्लुएंजा

निदान

नैदानिक प्रस्तुति

- लक्षण आमतौर पर 10–14 दिनों तक रहते हैं और स्व–सीमित होते हैं
- मुख्य लक्षण है नाक से पानी बहना, नाक में रूकावट या बंद होना, एकतरफा दांत या चेहरे में दर्द, चेहरे का भरा होना या दबाव, और कभी–कभी खांसी
- दर्द का स्थान शामिल साइनस पर निर्भर करता है
- तीव्र बैक्टीरिया साइनसाइटिस का संदेह तब होता है जब:
  – संकेत/लक्षण सुधार के बिना ≥10 दिनों तक बने रहते हैं या
  –प्रारंभिक हल्के चरण के बाद लक्षणों का महत्वपूर्ण रूप से बिगड़ना

माइक्रोबायोलॉजी परीक्षण

आमतौर पर जरूरत नहीं होती

अन्य प्रयोगशाला परीक्षण

आमतौर पर जरूरत नहीं होती

इमेजिंग

आमतौर पर जरूरत नहीं होती

इलाज

कोई एंटीबायोटिक देखभाल नहीं

- जब लक्षण <10 दिनों तक मौजूद हों और सुधार हो रहा हो
- रोगसूचक उपचार में ज्वरनाशक और एनाल्जेसिक दवाएं, खारे घोल से नाक की सिंचाई और सामयिक इंट्रानैसल शामिल है

ग्लुकोकोर्टिकोइड्स या डिकॉन्गेस्टेंटस

आईब्रुप्रोफेन 200–400 मिलीग्राम क्यू6–8एच (अधिकतम 2.4 ग्राम/दिन)

——————————या——————————

पैरासिटामोल (एसिटामिनोफेन) 500 मिलीग्राम–1 ग्राम क्यू4–6एच (अधिकतम 4 ग्राम/दिन)

- हेपेटिक हानि/सिरोसिस: अधिकतम 2 ग्राम/दिन

नैदानिक विचार

एंटीबायोटिक पर विचार किया जाना चाहिए यदि:
- लक्षणों की गंभीर शुरूआत
- बुखार ≥102°F) डिग्री फारेनहाइट और नाक से शुद्ध स्राव या लगातार कम से कम 3–4 दिनों तक चेहरे पर दर्द

एंटीबायोटिक इलाज

अधिकांश मामलों में एंटीबायोटिक उपचार की आवश्यकता नहीं होती है (जब एंटीबायोटिक का संकेत दिया जा सकता है तो ''नैदानिक विचार'' देखें)

अमोक्सिसिलिन 1 ग्राम क्यू8एच ओरल

या

अमोक्सिसिलिन क्लैवुलैनीक एसिड 500 मिलीग्राम 125 मिलीग्राम क्यू8एच ओरल

उपचार की अवधि: 5 दिन

तीव्र साइनस

बच्चे

परिभाषा

पेरानेसल साइनस और नाक गुहा की एक लक्षणात्मक सूजन। वयस्कों की तुलना में बहुत कम आम है क्योंकि साइनस पूरी तरह से विकसित नहीं होते है।

सर्वाधिक संभावित रोगजनक

श्वसन विषाणु:
- इन्फ्लूएंजा विषाणु (ए और बी)
- राइनो वायरस
- कोरोना वायरस (SARS-CoV-2 सहित)
- रेस्पिरेटरी सिंकाइटियल वायरस (आरएसबी)

बैक्टीरिया (शायद ही कभी)
- स्ट्रेपटोकोककस न्यूमोनिया
- हमोफिलिअस इन्फ्लुएंजा

निदान

नैदानिक प्रस्तुति

- लक्षण आमतौर पर 10–14 दिनों तक रहते हैं और स्व–सीमित होते हैं
- मुख्य लक्षण है नाक से पानी बहना, नाक में रूकावट या बंद होना, एकतरफा दांत या चेहरे में दर्द, चेहरे का भरा होना या दबाव, और कभी–कभी खांसी
- दर्द का स्थान शामिल साइनस पर निर्भर करता है
- तीव्र बैक्टीरिया साइनसाइटिस का संदेह तब होता है जब:
  – संकेत/लक्षण सुधार के बिना ≥10 दिनों तक बने रहते हैं या
  –प्रारंभिक हल्के चरण के बाद लक्षणों का महत्वपूर्ण रूप से बिगड़ना

माइक्रोबायोलॉजी परीक्षण

आमतौर पर जरूरत नहीं होती

अन्य प्रयोगशाला परीक्षण

आमतौर पर जरूरत नहीं होती

इमेजिंग

आमतौर पर जरूरत नहीं होती

इलाज

कोई एंटीबायोटिक देखभाल नहीं

- जब लक्षण <10 दिनों तक मौजूद हो और सुधार हो रहा हो
- रोगसूचक उपचार में ज्वरनाशक और एनाल्जेसिक दवाएं, खारे घोल से नाक की सिंचाई और सामयिक इंट्रानैसल शामिल है
ग्लुकोकोट्रिकोइड्स या डिकॉन्गेस्टेंटस

नैदानिक विचार

एंटीबायोटिक पर विचार किया जाना चाहिए यदि:
- लक्षणों की गंभीर शुरूआत
- बुखार ≥102 डिग्री फारेनहाइट और नाक से शुद्ध स्राव या लगातार कम से कम 3–4 दिनों तक चेहरे पर दर्द

Antibiotic Treatment Duration

5 दिन

एंटीबायोटिक इलाज

अधिकांश मामलों में एंटीबायोटिक उपचार की आवश्यकता नहीं होती है (जब एंटीबायोटिक का संकेत दिया जा सकता है है तो "नैदानिक विचार" देखें)

ACCESS
अमोक्सिसिलिन 80–90 मिलीग्राम/ किग्रा/दिन
ओरल वजन बैंड

3-<6 किग्रा	250 मिलीग्राम q12h
6-<10 किग्रा	375 मिलीग्राम q12h
10-<15 किग्रा	500 मिलीग्राम q12h
15-<20 किग्रा	750 मिलीग्राम q12h
≥ 20 किग्रा	500 मिलीग्राम q8h<br>या 1 ग्रा q12h

———————————या———————————

ACCESS
अमोक्सिसिलिन 80–90 मिलीग्राम/ किग्रा/दिन ओरल
ओरल वजन बैंड

3-<6 किग्रा	250 मिलीग्राम एमोक्स/खुराक q12h
6-<10 किग्रा	375 मिलीग्राम एमोक्स/खुराक q12h
10-<15 किग्रा	500 मिलीग्राम एमोक्स/खुराक q12h
15-<20 किग्रा	750 मिलीग्राम एमोक्स/खुराक q12h
≥ 20 किग्रा	500 मिलीग्राम एमोक्स/खुराक q8h<br>या 1 ग्रा एमोक्स/खुराक q12h

लक्षणात्मक इलाज

आईब्रुप्रोफेन (3 महीने से कम उम्र होने पर इसका उपयोग न करें)
•**दर्द नियंत्रण/ज्वरनाशक:** 5–10 मिलीग्राम/किग्रा क्यू6–8एच
• **ओरल वजन बैंड**

6-<10 किग्रा	50 मिलीग्राम q8h
10-<15 किग्रा	100 मिलीग्राम q8h
15-<20 किग्रा	150 मिलीग्राम q8h
20-<30 किग्रा	200 मिलीग्राम q8h
≥ 30 किग्रा	200–400 मिलीग्राम q6-8h

———————————या———————————

पैरासिटामोल (एसिटामिनोफेन)
•**दर्द नियंत्रण/ज्वरनाशक:** 10–15 मिलीग्राम/किग्रा क्यू6एच
• **ओरल वजन बैंड**

3-<6 किग्रा	60 मिलीग्राम q6h
6-<10 किग्रा	100 मिलीग्राम q6h
10-<15 किग्रा	150 मिलीग्राम q6h
15-<20 किग्रा	200 मिलीग्राम q6h
20-<30 किग्रा	300 मिलीग्राम q6h
≥ 30 किग्रा	500 मिलीग्राम q6-8h<br>(यकृत हानि/सिरोसिस होने पर अधिकतम 4 ग्राम/दिन या 2 ग्राम/दिन

तीव्र ओटिटिस मीडिया

वयस्क

परिभाषा

मध्य कान का संक्रमण जो वयस्कों में दुर्लभ है, अक्सर ऊपरी श्वसन पथ के वायरल संक्रमण की जटिलता के रूप में होता है।

निदान

नैदानिक प्रस्तुति

कान में दर्द की तीव्र शुरूआत (एकतरफा या द्विपक्षीय), बुखार ≥100.4 डिग्री फारेनहाइट) +/- कान बहना

माइक्रोबायोलॉजी परीक्षण

जरूरत नहीं

अन्य प्रयोगशाला परीक्षण

जरूरत नहीं

इमेजिंग

जरूरत नहीं

ऑटोस्कॉपी

यदि उपलब्ध हो तो निश्चित निदान के लिए आवश्यक है: उभरी हुई, सूजी हुई/संकुलित कर्णपटह झिल्ली

सर्वाधिक संभावित रोगजनक

श्वसन संबंधी विषाणु (ज्यादातर मामले):
- रेस्पिरेटरी सिंकाइटियल वायरस (आरएसवी)
- राइनो वायरस
- कोरोना वायरस (SARS-CoV-2 सहित)
- इन्फ्लूएंजा विषाणु (ए और बी)

जीवाणु (शायद ही कभी बैक्टीरिया सुपरइन्फेक्शन हो सकता है)
- स्ट्रेप्टोकोककस न्यूमोनिया
- हमोफिलिअस इन्फ्लुएंजा
- मोरक्सेला केटैराहेल्स
- स्ट्रेपटोकोकस पीजीसि (गुप ए स्ट्रेपटोकोकस)

इलाज

नैदानिक विचार

महत्वपूर्ण: अधिकांश मामले को लक्षणात्मक रूप से संबंधित किया जा सकता है **कोई एंटीबायोटिक उपचार नहीं**

एंटीबायोटिक पर विचार किया जाना चाहिए यदि:
- गंभीर लक्षण (उदाहरण के लिए, प्रणालीगत रूप से बहुत अस्वस्थ एनाल्जेसिक के बावजूद काम में दर्द, बुखार ≥102°F

लक्षणात्मक इलाज

आईब्रुप्रोफेन 200–400 मिलीग्राम क्यू6–8एच (अधिकतम 2.4 ग्राम/दिन)

पैरासिटामोल (एसिटामिनोफेन) 500 मिलीग्राम– 1 ग्राम क्यू4–6एच (अधिकतम 4 ग्राम/दिन)
- **यकृत हानि/सिरोसिस:** अधिकतम 2 ग्राम/दिन

एंटीबायोटिक उपचार की अवधि

5 दिन

एंटीबायोटिक इलाज

पहली पसंद

ACCESS अमोक्सिसिलीन 500 मिलीग्राम क्यू8एच **ओरल**

दुसरी पसंद

ACCESS अमोक्सिसिलीन + क्लैवुलैनीक एसिड 500 मिलीग्राम + 125 मिलीग्राम क्यू8एच **ओरल**

स्त्रोत: WHO AWaRe पुस्तक पर आधारित

तीव्र ओटिटिस मीडिया

बच्चे

परिभाषा

मध्य कान का संक्रमण जो ज्यादातर 5 वर्ष से कम उम्र के बच्चें में होता है, अक्सर ऊपरी श्वसन पथ के वायरल संक्रमण की जटिलता के रूप में होता है।

सर्वाधिक संभावित रोगजनक

श्वसन विषाणु:
- रेस्पिरेटरी सिंकाइटियल वायरस (आरएसवी)
- राइनो वायरस
- कोरोना वायरस (SARS-CoV-2 सहित)
- इन्फ्लूएंजा विषाणु (ए और बी)

जीवाणु (शायद ही कभी बैक्टीरिया सुपरइन्फेक्शन हो सकता है):
- स्ट्रेपटोकोकस न्यूमोनिया
- हमोफिलिअस इन्फ्लुएंजा
- मोरक्सेला केटैराहेल्स
- स्ट्रेपटोकोकस पीजीसि (गुप ए स्ट्रेपटोकोकस)

निदान

नैदानिक प्रस्तुति

कान में दर्द की तीव्र शुरूआत (एकतरफा या द्विपक्षीय), बुखार ≥100.4 डिग्री फारेनहाइट) +/- कान बहना

माइक्रोबायोलॉजी परीक्षण

जब तक जरूरत नहीं होती

अन्य प्रयोगशाला परीक्षण

जब तक जरूरत नहीं होती

इमेजिंग

जब तक जरूरत नहीं होती

ऑटोस्कॉपी

यदि उपलब्ध हो तो निश्चित निदान के लिए आवश्यक है: उभरी हुई, सूजी हुई / संकुलित कर्णपटह झिल्ली

स्त्रोत: WHO AWaRe पुस्तक पर आधारित

तीव्र ओटिटिस मीडिया

℞ इलाज

नैदानिक विचार

महत्वपूर्ण: अधिकांश मामले को बिना किसी एंटीबायोटिक उपचार के लक्षणात्मक रूप में प्रबंधित किया जा सकता है, विशेषकर 2 वर्ष से अधिक उम्र के बच्चों में

एंटीबायोटिक पर विचार किया जाना चाहिए यदि:

- गंभीर वायरस (उदाहरण के लिए, प्रणालीगत रूप में बहुत अस्वस्थ, एनाल्जैसिक के बावजूद कान में दर्द, बुखार ≥102°F)
- 2 वर्ष से कम उम्र के बच्चों में द्विपक्षीय तीव्र ओटिटिस मीडिया

℞ लक्षणात्मक इलाज

आईबुप्रोफेन (3 महीने से कम उम्र होने पर इसका उपयोग न करें)
- **दर्द नियंत्रण / ज्वरनाशक:** 5–10 मिलीग्राम / किग्रा क्यू6–8 एच
- **ओरल वजन बैंड**

6-<10 किग्रा	50 मिलीग्राम q8h
10-<15 किग्रा	100 मिलीग्राम q8h
15-<20 किग्रा	150 मिलीग्राम q8h
20-<30 किग्रा	200 मिलीग्राम q8h
≥ 30 किग्रा	200–400 मिलीग्राम q6-8h (या 2.4 ग्रा / दिन)

——————————या——————————

पैरासिटामोल (एसिटामिनोफेन)
- **दर्द नियंत्रण / ज्वरनाशक:** 5–10 मिलीग्राम / किग्रा क्यू6 एच
- **ओरल वजन बैंड**

3-<6 किग्रा	60 मिलीग्राम q6h
6-<10 किग्रा	100 मिलीग्राम q6h
10-<15 किग्रा	150 मिलीग्राम q6h
15-<20 किग्रा	200 मिलीग्राम q6h
20-<30 किग्रा	300 मिलीग्राम q6h
≥ 30 किग्रा	500 मिलीग्राम q4-6h (यकृत हानि / सिरोसिस होने पर अधिकतम 4 ग्रा / दिन या 2 ग्राम / दिन)

एंटीबायोटिक इलाज की अवधि

5 दिन

℞ एंटीबायोटिक इलाज

पहली पसंद

ACCESS

एमोक्सिसीलीन 80–90 मिलीग्राम / किग्रा / दिन **ओरल**
- **ओरल वजन बैंड**

3-<6 किग्रा	250 मिलीग्राम q12h
6-<10 किग्रा	375 मिलीग्राम q12h
10-<15 किग्रा	500 मिलीग्राम q12h
15-<20 किग्रा	750 मिलीग्राम q12h
≥ 20 किग्रा	500 मिलीग्राम q8h (या 1 ग्रा q12h)

Second Choice

ACCESS

एमोक्सिसीलीन + क्लेवुलैनिक 80–90 मिलीग्राम / किग्रा / दिन एमोक्सिसिलिन घटक **ओरल**
- **ओरल वजन बैंड**

3-<6 किग्रा	250 मिलीग्राम एमोक्स / खुराक q12h
6-<10 किग्रा	375 मिलीग्राम एमोक्स / खुराक q12h
10-<15 किग्रा	500 मिलीग्राम एमोक्स / खुराक q12h
15-<20 किग्रा	750 मिलीग्राम एमोक्स / खुराक q12h
≥ 20 किग्रा	500 मिलीग्राम एमोक्स / खुराक q8h या 1 ग्रा एमोक्स / खुराक q12h)

तीव्र ओटिटिस मीडिया

वयस्क

परिभाषा

ग्रसनी की सूजन, जिसमें गले में खराश और निगलने में दर्द होता है।

सर्वाधिक संभावित रोगजनक

वायरस (>80% मामले)

जीवाणु:

• ग्रुप ए स्ट्रेप्टोकोककस (5–10% वयस्कों में)

निदान

नैदानिक प्रस्तुति

गले में खराश और निगलने में दर्द

• **वायरस:** लक्षण अक्सर वायरल ऊपरी सतह श्वसन पथ संक्रमण (यूआरटीआइ) के समान ही होते हैं खांसी, सिरदर्द और मायालगिया

• **जीवाणु:** अधिक गंभीर रूप से, बुखार (≥100.4 डिग्री फारेनहाइट), कोमल ग्रीवा लिम्फ और ग्रसनी स्राव

माइक्रोबायोलॉजी परीक्षण

• परीक्षण की आमतौर पर आवश्यकता नहीं होती

• यदि उपलब्ध हो–ग्रुप ए के लिए थ्रोट कल्चर या रैपिड एंटीजन टेस्ट स्ट्रैपटोकोकस

अन्य प्रयोगशाला परीक्षण

रक्त परीक्षण की आमतौर पर आवश्यकता नहीं होती

इमेजिंग

आमतौर पर आवश्यकता नहीं होती

अन्न नलिका का रोग

वयस्क

पेज 2 का 2

सैटोर क्लिनिकल स्कोरिंग सिस्टम

• यह प्रणाली संक्रमण की उत्पत्ति (जीवाणु या वायरल) को इंगित करने में मदद कर सकती है और यह भी बता सकती है कि एंटीबायोटिक आवश्यक है या नहीं।

संकेत एवं लक्षण
• बुखार >38.0 डिग्री फारेनहाइट
• कोई खांसी नहीं
• कोमल पूर्वकाल ग्रीवा लसीकाप्रवशोथ
• टॉन्सिल स्रावित होता है

स्कोर 0–2
• एंटीबायोटिक की आवश्यक नहीं।

स्कोर 3–4
• एंटीबायोटिक उपचार की सिफारिश की गई

℞ इलाज

℞ लक्षाणात्मक इलाज

आईबुप्रोफेन 200–400 मिलीग्राम क्यु6–8एच (अधिकतम 2.4 ग्राम/दिन

———————या——————

पैरासिटामोल (एसिटामिनोफेस) 500 मिलीग्राम–1 ग्राम क्यू4–6 एच (अधिकतम 4 ग्राम/दिन
• **यकृत हानि/सिरोसिस:** अधिकतम 2ग्राम/दिन

एंटीबायोटिक उपचार की अवधि

• एमोक्सिसिलिन और फेनोसिमिथाइलपेनिसिलिन 10 दिन
• सेफालेक्सिन 5 दिन

℞ एंटीबायोटिक उपचार

पहली पसंद

ACCESS — एमोक्सिसिलिन 500 मिलीग्राम क्यू8एच **ओरल**

———————————या———————————

ACCESS — फेनोक्सीमीथीसपेनीसिलीन (एज पोटेशियम) 500 मिलीग्राम (800 000 आईयू) क्यू6एच **ओरल**

दुसरी पसंद

ACCESS — सेफौलेक्सिन 500 मिलीग्राम क्यू8एच **ओरल**

WATCH

अन्न नलिका का रोग

बच्चे

परिभाषा

ग्रसनी की सूजन, जिसमें गले में खराश और निगलने में दर्द होता है

सर्वाधिक संभावित रोगजनक

वायरस (>80% मामले)

जीवाणु:

• ग्रुप ए स्ट्रेपटोकोककस (20–30% बच्चों में)

निदान

नैदानिक प्रस्तुति

गले में खराश और निगलने में दर्द

• **वायरस:** लक्षण अक्सर खांसी के साथ वायरल ऊपरी श्वसन संक्रमण (यूआरटीआई) के समान ही होते हैं सिरदर्द और मायालगिया

• **जीवाणु:** अधिक गंभीर रूप से, बुखार (≥100.4 डिग्री फारेनहाइट), कोमल ग्रीवा लिम्फ और ग्रसनी स्राव

माइक्रोबायोलॉजी परीक्षण

• परीक्षण की आमतौर पर आवश्यकता नहीं होती

• यदि उपलब्ध हो–ग्रुप ए के लिए थ्रोट कल्चर या रैपिड एंटीजन टेस्ट स्ट्रैपटोकोकस

अन्य प्रयोगशाला परीक्षण

रक्त परीक्षण की आमतौर पर जरूरत नहीं होती

इमेजिंग

आमतौर पर जरूरत नहीं होती

बच्चे

अन्न नलिका का रोग

सैटोर क्लिनिकल स्कोरिंग सिस्टम

• यह प्रणाली संक्रमण की उत्पत्ति (जीवाणु या वायरल) को इंगित करने में मदद कर सकती है और यह भी बता सकती है कि एंटीबायोटिक आवश्यक है या नहीं।

संकेत एवं लक्षण (प्रत्येक 1 अंक)
• बुखार >38.0 डिग्री फारेनहाइट
• कोई खांसी नहीं
• कोमल पूर्वकाल ग्रीवा लसीकाप्रवशोथ
• टॉन्सिल स्रावित होता है

स्कोर 0–2
• एंटीबायोटिक की आवश्यक नहीं।

स्कोर 3–4
• एंटीबायोटिक पुनमुल्यांकन की अनुशंसा की गई

इलाज

लक्षणात्मक इलाज

आईबुप्रोफेन (3 महीने से कम उम्र होने पर इसका उपयोग न करें)
• **दर्द नियंत्रण / ज्वरनाशक:** 5–10 मिलीग्राम / किग्रा क्यू6–8 एच
• **ओरल वजन बैंड**

6-<10 किग्रा	50 मिलीग्राम q8h
10-<15 किग्रा	100 मिलीग्राम q8h
15-<20 किग्रा	150 मिलीग्राम q8h
20-<30 किग्रा	200 मिलीग्राम q8h
≥ 30 किग्रा	200–400 मिलीग्राम q6-8h (अधिकतम 2.4 ग्रा / दिन)

————————–या–————————

पैरासिटामोल (एसिटामिनोफेन)
• **दर्द नियंत्रण / ज्वरनाशक:** 10–15 मिलीग्राम / किग्रा क्यू6 एच
• **ओरल वजन बैंड**

3-<6 किग्रा	60 मिलीग्राम q6h
6-<10 किग्रा	100 मिलीग्राम q6h
10-<15 किग्रा	150 मिलीग्राम q6h
15-<20 किग्रा	200 मिलीग्राम q6h
20-<30 किग्रा	300 मिलीग्राम q6h
≥ 30 किग्रा	500 मिलीग्राम q4-6h (यकृत हानि / सिरोसिस होने पर अधिकतम 4 ग्रा / दिन या 2 ग्राम / दिन)

एंटीबायोटिक इलाज की अवधि

• एमोक्सिसिलिन और फेनोक्सिमिथाइलपेनिसिलिन 10 दिन
• सेफालेक्सिन 5 दिन

एंटीबायोटिक इलाज

पहली पसंद

ACCESS एमोक्सिसीलीन 80–90 मिलीग्राम / किग्रा / दिन **ओरल**
• **ओरल वजन बैंड**

3-<6 किग्रा	250 मिलीग्राम q12h
6-<10 किग्रा	375 मिलीग्राम q12h
10-<15 किग्रा	500 मिलीग्राम q12h
15-<20 किग्रा	750 मिलीग्राम q12h
≥ 20 किग्रा	500 मिलीग्राम q8h (या 1 ग्रा q12h)

———————–या–———————

ACCESS फेनोक्सीमीथीपेनीसिलीन (एज पोटैशियम): 10–15 मिलीग्राम / किग्रा / खुराक (16 000–24 000 आईयू / किग्रा / खुराक) क्यू6–8एच **ओरल**

दुसरी पसंद

ACCESS सेफालेक्सीन 25 मिलीग्राम / किग्रा / खुराक **ओरल**
• **ओरल वजन बैंड**

3-<6 किग्रा	125 मिलीग्राम q12h
6-<10 किग्रा	250 मिलीग्राम q12h
10-<15 किग्रा	375 मिलीग्राम q12h
15-<20 किग्रा	500 मिलीग्राम q12h
20-<30 किग्रा	625 मिलीग्राम q12h
≥ 30 किग्रा	500 मिलीग्राम q8h

स्त्रोत: WHO AWaRe पुस्तक पर आधारित
