# Supplementary material for: Impact of WHO AWaRe Antibiotic Handbook training on antibiotics prescribing knowledge among private primary care providers: a vignette-based, prep–post pilot study in Patna, India
Source: Antimicrob Resist Infect Control. 2026 Mar 27;15:67. doi: 10.1186/s13756-026-01735-6 (PMC13151071; doi:10.1186/s13756-026-01735-6)
Supplement: Supplementary file 2 — Supplementary Material 2 [file 13756_2026_1735_MOESM2_ESM.pdf]

Endline survey questionnaire: **Participant ID :**

1. Was the training session (done by Sumanth sir) on the WHO antibiotics handbook useful for you?
    - Not at all useful
    - Slightly useful
    - Moderately useful
    - Very useful

2. Please briefly explain the reason for your response (on the training session) above.
   [Note: PLEASE WRITE AS SAID BY THE PARTICIPANT AND WRITE IT CLEARLY]

   1) ……

   ………

   2) ……

   ……

   3) ……

   ……

3. Was the printed WHO Antibiotics handbook (for formal providers) or infographic (for informal providers) useful for you?
    - Not at all useful
    - Slightly useful
    - Moderately useful
    - Very useful

4. Please briefly explain the reason for your response (handbook or infographic) above.
   [Note: PLEASE WRITE AS SAID BY THE PARTICIPANT AND WRITE IT CLEARLY]

   1) ……

   ………

   2) ……

   ……

   3) ……

   ……

5. Was the Firstline Application (mobile phone version of the antibiotics handbook) useful for you?
    - Not at all useful
    - Slightly useful
    - Moderately useful
    - Very useful

6. Please briefly explain the reason for your response (mobile version of the antibiotic handbook) above.
[Note: PLEASE WRITE AS SAID BY THE PARTICIPANT AND WRITE IT CLEARLY]

1) ………

……

2) ………

………

3) ………

………

7. Would you recommend your peers/colleagues to participate in this program?
- Yes
- No
- Maybe

8. Do you have any suggestions on how this program can be improved?
[Note: PLEASE WRITE AS SAID BY THE PARTICIPANT AND WRITE IT CLEARLY]

1) ………

……

2) ………

………

3) ………

………

9. Any other comments you would like to share?
[Note: PLEASE WRITE AS SAID BY THE PARTICIPANT AND WRITE IT CLEARLY]

1) ………

……

2) ………

………

3) ………

………

THANK YOU FOR YOUR PARTICIPATION!
